# Longevity Humans Have Youthful Erythrocyte Function and Metabolic Signatures

**DOI:** 10.1111/acel.14482

**Published:** 2025-02-09

**Authors:** Fang Yu, Changhan Chen, Wuping Liu, Zhixiang Zhao, Yuhua Fan, Zhenjiang Li, Weilun Huang, Tingting Xie, Cheng Luo, Zhouzhou Yao, Qi Guo, Zhiyu Yang, Juan Liu, Yujin Zhang, Rodney E. Kellems, Jian Xia, Ji Li, Yang Xia

**Affiliations:** ^1^ Department of Neurology Central South University Changsha China; ^2^ National Medical Metabolomics International Collaborative Research Center Central South University Changsha China; ^3^ National Clinical Research Center for Geriatric Disorders Central South University Changsha China; ^4^ Hunan Key Laboratory of Aging Biology Central South University Changsha China; ^5^ Department of Dermatology Central South University Changsha China; ^6^ Department of Otolaryngology Head and Neck Surgery Central South University Changsha China; ^7^ Department of General Medicine, Xiangya Hospital Central South University Changsha China; ^8^ Department of Biochemistry and Molecular Biology The University of Texas McGovern Medical School at Houston Houston Texas USA

**Keywords:** erythrocyte, longevity, metabolomic, oxidative stress, oxygen release

## Abstract

Longevity individuals have lower susceptibility to chronic hypoxia, inflammation, oxidative stress, and aging‐related diseases. It has long been speculated that “rejuvenation molecules” exist in their blood to promote extended lifespan. We unexpectedly discovered that longevity individuals exhibit erythrocyte oxygen release function similar to young individuals, whereas most elderly show reduced oxygen release capacity. Untargeted erythrocyte metabolomics profiling revealed that longevity individuals are characterized by youth‐like metabolic reprogramming and these metabolites effectively differentiate the longevity from the elderly. Quantification analyses led us to identify multiple novel longevity‐related metabolites within erythrocytes including adenosine, sphingosine‐1‐phosphate (S1P), and glutathione (GSH) related amino acids. Mechanistically, we revealed that increased bisphosphoglycerate mutase (BPGM) and reduced MFSD2B protein levels in the erythrocytes of longevity individuals collaboratively work together to induce elevation of intracellular S1P, promote the release of glyceraldehyde‐3‐phosphate dehydrogenase (GAPDH) from membrane to the cytosol, and thereby orchestrate glucose metabolic reprogramming toward Rapoport–Luebering Shunt to induce the 2,3‐BPG production and trigger oxygen delivery. Furthermore, increased glutamine and glutamate transporter expression coupled with the enhanced intracellular metabolism underlie the elevated GSH production and the higher anti‐oxidative stress capacity in the erythrocytes of longevity individuals. As such, longevity individuals displayed less systemic hypoxia‐related metabolites and more antioxidative and anti‐inflammatory metabolites in the plasma, thereby healthier clinical outcomes including lower inflammation parameters as well as better glucose–lipid metabolism, and liver and kidney function. Overall, we identified that youthful erythrocyte function and metabolism enable longevity individuals to better counteract peripheral tissue hypoxia, inflammation, and oxidative stress, thus maintaining healthspan.

## Graphical abstract

Longevity humans have “a youthful erythrocyte function and metabolism” with a youth‐like oxygen release and anti‐oxidative capability to combat tissue hypoxia and improve healthspan.

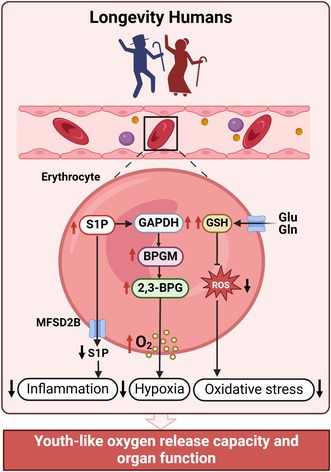

## 1 Introduction

Individuals who live past the age of 90 are defined as longevity individuals and are examples of highly successful aging, often referred to as increased healthspan. These individuals are equipped with a better capability to counteract chronic tissue hypoxia, inflammation, and oxidative stress (Engberg et al. 2009; Zhu et al. 2023a; Belenguer‐Varea et al. 2020) and thus a lower susceptibility to age‐related diseases including cardiovascular disease and Alzheimer's disease (Campisi et al. 2019; Guo et al. 2022). Such an advantage makes longevity individuals an ideal population for the investigation of cellular and molecular mechanisms underlying better aging with the ultimate goal of promoting lifespan and healthspan and decreasing the burden of degenerative diseases with important social and economic benefits.

Humans have long been fascinated with longevity. Substantial effort and research have been devoted to understanding the mechanisms underlying longevity, searching for circulating rejuvenation molecules that prolong lifespan. Currently, high‐throughput multi‐omics has revealed that longevity is a complex trait in which genetic, epigenetic, and environmental determinants interact with each other contributing to better aging and longer lifespan (Dato et al. 2017). For example, genetic studies indicate that genes associated with longevity contribute to only 20%–30% of the underlying processes (Caruso et al. 2022). Notably, the most consistent evidence has been obtained for variants in *APOE* and *FOXO3A* genes using either genome‐wide association studies (GWAS) or candidate gene studies (van den Berg et al. 2019). Epigenetically, sirtuins, nicotinamide adenine dinucleotide (NAD^+^)‐dependent protein deacetylases, are well known for their role in epigenetic modification and longevity (Wątroba et al. 2017; Korotkov, Seluanov, and Gorbunova 2021). They control the activity of several important proteins associated with the aging process including NF‐κB, p53, and FOXO. Molecularly, the protein Klotho, which declines during aging, is a longevity factor that combats aging oxidative stress and inflammation and thereby improves cognition and extends healthspan and lifespan (Abraham and Li 2022). For example, Klotho administration increases the levels of platelet factor 4 (PF4) and rejuvenates the aged brain (Park et al. 2023). Additionally, insulin‐like growth factor‐1 (IGF‐1) (Junnila et al. 2013), mammalian target of rapamycin (mTOR) (Wei et al. 2015), and AMP‐activating protein kinase (AMPK) (Weir et al. 2017) are well‐recognized molecules in longevity. At the microbiome level, the gut microbiota and gut‐derived metabolites have also been recognized as important contributors to longevity (Cӑtoi et al. 2020; Pang et al. 2023; Sato et al. 2021). Distinct gut microbiota such as Akkermansia, Bifidobacterium, and Christensenellaceae are associated with longevity (Kong et al. 2019; Biagi et al. 2016). Notably, a recent study reported that the gut microbiota of centenarians exhibits youth‐similar features (Pang et al. 2023). However, the molecular and metabolic mechanisms underlying longevity remain poorly understood.

Intriguingly, most of the 12 hallmarks of aging such as “deregulated nutrient sensing,” “mitochondrial dysfunction,” “dysbiosis,” and “microbiome,” as well as “epigenetics” are closely linked with metabolic changes and metabolite‐mediated posttranslational modification including methylation, acetylation, and phosphorylation, suggesting that metabolic reprogramming plays important roles in longevity to promote lifespan. It is worth noting that longevity blue zones are geographical areas of the planet harboring a measurably higher proportion of long‐lived individuals compared with the average number of longevity individuals living elsewhere. Multiple studies related to the Blue Zones (e.g., Okinawa, Sardinia, and Costa Rica, among others) highlight lifestyle and diet strongly linked to a long lifespan. These findings suggest that metabolic differences have the potential to influence the aging process and longevity (Appel 2008). Supporting this hypothesis, early studies identified various metabolites including amino acids (AAs), phospholipids, nucleosides, bile acids, short‐chain fatty acids, and polyamines associated with longevity (Sato et al. 2021; Zhou, Hu, and Wang 2021; Cai et al. 2022; Eisenberg et al. 2016; López‐Otín et al. 2016; Cheng et al. 2015; Wang et al. 2023). Although human translational studies have evaluated the efficacy of NAD^+^ precursors and spermidine (Guarente, Sinclair, and Kroemer 2024), it is disappointing that the outcomes of the current treatments and the ongoing trials remain suboptimal or controversial. This unfortunate situation stems from a poor understanding of the cellular, and molecular mechanisms underlying the nature of longevity. There is a major need to conduct longevity‐related research from a completely new angle.

Notably, it has been long speculated that “rejuvenation molecules” exist in the blood of younger individuals that promote better aging and a longer lifespan in longevity individuals. For example, heterochronic parabiosis (in which the circulatory systems of young and aged animals are joined) can reverse age‐related impairments in aged mice (Ma et al. 2022; Zhang et al. 2023), providing proof‐of‐principle evidence of rejuvenating cells and molecules existing in the blood to counteract aging. Early studies largely attributed this rejuvenating effect to stem cells and extracellular vesicles (Conboy et al. 2005; Ribaudo and Gianoncelli 2023). Erythrocytes as the most abundant and only cell type carrying and delivering oxygen (O_2_) play a vital role in orchestrating energy metabolism, anti‐oxidative stress, and survival of every single tissue within our body (Bianconi et al. 2013). Notably, emerging evidence underscores that erythrocyte spermidine is a highly heritable phenotype associated with longevity (Kaminsky et al. 2024), while the fatty acid component in erythrocyte membrane has potential as a biomarker for longevity (Puca et al. 2008). Moreover, erythrocyte membranes from centenarians exhibit distinct characteristics compared to elderly individuals, potentially offering protection against cellular damage (Rabini et al. 2002). However, a comprehensive analysis of erythrocyte function, metabolism, and their interactions with plasma remains largely unexplored.

To address the potential role of erythrocytes in longevity, we made a substantial effort to precisely assess the erythrocyte oxygen‐releasing capability, followed by comprehensive metabolomic profiling of erythrocytes and plasma and clinical outcome association analyses in a large cross‐sectional cohort human study of 730 participants encompassing longevity individuals (≥ 90 years) and diverse age groups. From this analysis we expect to (i) identify erythrocyte‐specific functional changes, (ii) define specific “youth‐like and longevity metabolic signatures” in erythrocytes, (iii) delineate their metabolic and molecular consequences, (iv) probe the crosstalk between erythrocytes and plasma, and (v) differentiate the longevity based on newly identified longevity and youth‐like metabolites. We report here that erythrocytes have specific “longevity molecules and metabolites” that support a “youth‐like” oxygen delivery capacity. As a result, “youth‐like erythrocytes” are more efficient in transporting O_2_ and better positioned to maintain optimal peripheral tissue oxygenation and sufficient energy supply as well as anti‐inflammation, anti‐oxidative stress, and anti‐tissue damage, thereby promoting anti‐aging and longevity.

## 2 Results

### 2.1 Study Design and Characteristics of the Study Participants

To assess the changes in erythrocyte function and metabolism and define the crosstalk between erythrocytes and overall plasma metabolism, we conducted a large cohort study to compare longevity individuals with young and old adults, respectively. Participant characteristics of the community‐dwelling individuals from Hunan Province with different age groups (*n* = 730) are presented in Table 1. All individuals were divided into four groups based on previous studies related to aging and longevity (Sato et al. 2021; Prospective Studies Collaboration et al. 2007; Rea et al. 2000): the longevity (L) group (90–102 years, *n* = 216), the elderly (E) group (70–89 years, *n* = 119), the middle‐aged (M) group (56–69 years, *n* = 216), and the young (Y) group (21–55 years, *n* = 179). Sex distribution among the four groups was well‐matched. Among these, the elderly group (mean age: 76 years) included individuals with ages approximating the average regional life expectancy of Hunan Province, most of whom are likely in their final decade, akin to the longevity group.

**TABLE 1:** Demographic, clinical, and laboratory characteristics of community‐dwelling individuals.

Variables	Y (21–55), *N* = 179	M (56–69), *N* = 216	E (70–89), *N* = 119	L (90–102), *N* = 216	*p*
Age (years)	45 ± 10	62 ± 4	76 ± 5	92 ± 3	< 0.001
Sex					
Male	87 (49%)	118 (55%)	62 (52%)	104 (48%)	0.500
Female	92 (51%)	98 (45%)	57 (48%)	112 (52%)	
RBC (10^12^/L)	4.57 (4.31–4.89)	4.54 (4.28–4.78)	4.46 (4.22–4.66)	3.93 (3.61–4.24)	< 0.001
Hb (g/L)	143 (134–151)	143 (135–152)	139 (132–148)	126 (116–136)	< 0.001
HCT (%)	42.8 (40.1–44.9)	42.8 (40.6–45.4)	41.9 (39.5–44.4)	38.2 (35.1–41.1)	< 0.001
MCV (fL)	92.9 (89.7–96.8)	95.3 (92.6–98.4)	94.8 (91.3–97.5)	97.6 (94.8–100.4)	< 0.001
MCH (pg)	31.10 (30.10–32.10)	31.60 (30.70–32.80)	31.50 (30.40–32.40)	32.10 (31.00–33.00)	< 0.001
MCHC (g/L)	336 (329–339)	332 (329–336)	333 (329–337)	328 (323–333)	< 0.001
RDWCV (%)	13.14 (12.40–13.75)	12.80 (12.28–13.40)	13.50 (12.90–14.25)	13.20 (12.80–13.70)	< 0.001
WBC (10^9^/L)	6.00 (5.10–7.08)	6.26 (5.50–7.21)	6.00 (5.36–7.05)	5.96 (5.12–7.21)	0.210
N (10^9^/L)	3.36 (2.80–4.18)	3.58 (2.96–4.38)	3.63 (3.20–4.32)	3.63 (2.95–4.41)	0.110
L (10^9^/L)	2.00 (1.62–2.40)	1.96 (1.62–2.32)	1.69 (1.40–2.20)	1.67 (1.31–2.07)	< 0.001
NLR	1.65 (1.37–2.16)	1.77 (1.36–2.38)	2.20 (1.76–2.70)	2.17 (1.61–2.86)	< 0.001
Plt (10^9^/L)	217 (178–254)	212 (189–237)	219 (182–261)	191 (158–218)	< 0.001
ALT (U/L)	20 (15–29)	19 (16–26)	18 (14–25)	12 (9–15)	< 0.001
AST (U/L)	25 (20–28)	25 (21–29)	24 (21–27)	24 (20–27)	0.270
TPA (g/L)	74.0 (71.6–76.6)	73.7 (71.3–76.5)	74.2 (72.3–77.1)	72.7 (68.5–76.1)	< 0.001
ALB (g/L)	44.9 (43.7–46.5)	44.8 (43.3–46.3)	45.1 (43.7–47.2)	42.3 (40.2–44.2)	< 0.001
GLOB (g/L)	29.2 (26.8–30.9)	28.9 (26.6–31.2)	29.0 (26.9–31.0)	30.1 (27.5–32.5)	0.005
AGR (μmol/L)	1.60 (1.40–1.70)	1.57 (1.40–1.70)	1.60 (1.40–1.70)	1.40 (1.30–1.50)	< 0.001
TBil (μmol/L)	12.8 (10.6–16.0)	12.6 (9.9–15.3)	13.5 (10.8–16.4)	11.1 (8.5–13.9)	< 0.001
DBil (μmol/L)	3.50 (2.69–4.50)	3.23 (2.40–4.16)	3.70 (2.88–5.00)	3.08 (2.29–4.34)	< 0.001
TBA (μmol/L)	4.0 (2.5–7.4)	4.2 (2.4–6.9)	3.5 (1.9–5.3)	5.8 (3.9–10.0)	< 0.001
Urea (mmol/L)	5.10 (4.38–6.06)	5.44 (4.52–6.56)	5.19 (4.41–6.22)	6.59 (5.41–8.11)	< 0.001
UA (μmol/L)	316 (276–381)	292 (251–354)	327 (258–380)	293 (249–350)	< 0.001
Creatinine (μmol/L)	74 (61–85)	70 (61–80)	74 (65–85)	75 (65–97)	< 0.001
eGFR (mL/min/1.73 m^2^)	97 (84–110)	84 (71–101)	92 (80–110)	66 (50–78)	< 0.001
Glucose (mmol/L)	5.14 (4.77–5.39)	5.14 (4.73–5.71)	5.34 (4.83–6.36)	5.12 (4.66–5.62)	0.007
TG (mmol/L)	1.57 (1.11–1.99)	1.40 (0.98–2.12)	1.32 (0.94–1.81)	1.26 (0.94–1.64)	< 0.001
TC (mmol/L)	5.42 (4.78–5.93)	5.51 (4.76–6.30)	5.32 (4.39–6.05)	5.24 (4.63–6.10)	0.120
HDL (mmol/L)	1.37 (1.17–1.60)	1.44 (1.24–1.66)	1.42 (1.15–1.71)	1.58 (1.33–1.86)	< 0.001
LDL (mmol/L)	3.17 (2.72–3.66)	3.09 (2.56–3.82)	3.06 (2.63–3.59)	2.72 (2.41–3.42)	< 0.001
BMI (kg/m^2^)	22.9 (20.8–24.8)	22.7 (20.0–25.0)	23.0 (21.5–26.2)	22.7 (20.7–25.2)	0.200
SBP (mmHg)	141 (120–157)	149 (130–170)	131 (116–146)	157 (140–174)	< 0.001
DBP (mmHg)	81 (74–90)	90 (78–97)	83 (74–91)	90 (80–98)	< 0.001

*Note:* Age, sex, blood cell parameters, liver and kidney function, blood lipids, BMI, and blood pressure values are presented. *p* Values indicate significant differences among the four groups. Continuous variables were compared using a two‐sided Kruskal–Wallis rank‐sum test, while categorical variables were analyzed using a two‐sided Fisher's exact test.

### 2.2 Unique Erythrocyte and Organ Function of Longevity Individuals

**FIGURE 1:**
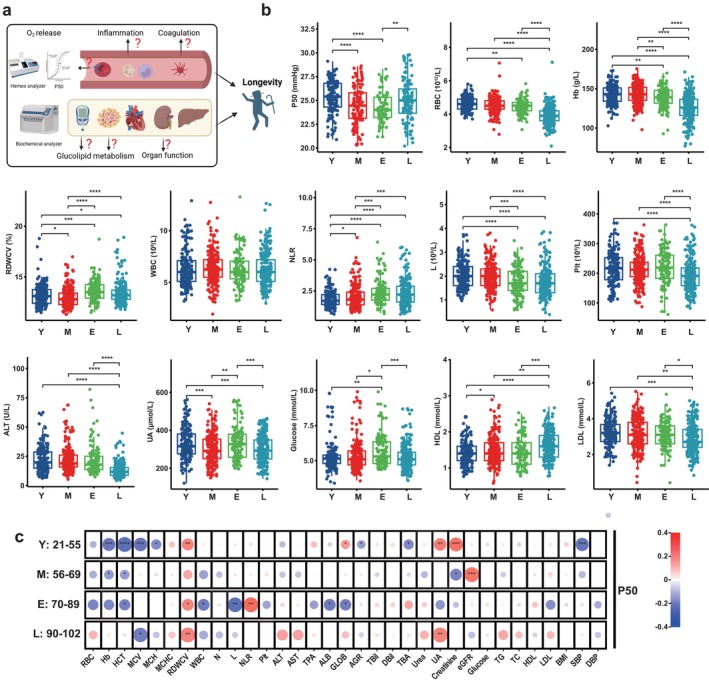
Unique oxygen release capacity and organ function of longevity individuals. (a) Schematic representation of the study of erythrocyte oxygen release capacity and clinical parameters in aging and longevity. (b) Blood tests from the longevity group (90–102 years, *n* = 216), the elderly group (70–89 years, *n* = 119), the middle‐aged group (56–69 years, *n* = 216), and the young group (21–55 years, *n* = 179). P50, the partial pressure of O_2_ required for 50% Hb binding to O_2_; ALT, alanine transaminase; Hb, hemoglobin concentration; HDL, high‐density lipoprotein cholesterol; L, lymphocyte; LDL, low‐density lipoprotein cholesterol; NLR, neutrophil–lymphocyte ratio; Plt, platelet; RBC, red blood cell count; RDWCV, red cell distribution width with the coefficient of variation; UA, uric acid; WBC, white blood cell count. Data are mean ± s.d. ****p < 0.0001, ***p < 0.001, **p < 0.01, *p < 0.05; Kruskal–Wallis with Dunn's test; ns, not significant. (c) Correlation between P50 and clinical variables in different age groups (red represents positive correlation and blue represents negative correlation). AGR, albumin‐globulin ratio; ALB, albumin; AST, aspartate aminotransferase; BMI, body mass index; DBil, direct bilirubin; DBP, diastolic blood pressure; eGFR, estimated glomerular filtration rate; GLOB, globulin; HCT, hematocrit; MCH, mean corpuscular hemoglobin; MCHC, mean corpuscular hemoglobin concentration; MCV, mean corpuscular volume; N, neutrophil; SBP, systolic blood pressure; TBA, total bile acid; TBil, total bilirubin; TC, total cholesterol; TG, triglyceride; TPA, total serum protein. ****p < 0.0001, ***p < 0.001, **p < 0.01, *p < 0.05; Spearman correlation analysis.

First, to define the functional changes of erythrocytes, we measured the oxygen release capacity of most of the participants by measuring P50 (the partial pressure of oxygen that provides 50% saturation of hemoglobin binding to O_2_) (illustration in Figure 1a). Surprisingly, we found that before the age of 89, P50 gradually decreased with age, while in the longevity group, the P50 level did not drop, but instead was maintained at a higher level than that of the 70–89 age group and comparable to the levels in young and middle‐aged groups (Figure 1b). The nonlinear fitting curve showed that P50 was progressively decreased from young to the elderly but maintained at a level close to young individuals in the longevity group in both genders (Figure S1a). Moreover, females exhibited a trend of relatively but not significantly higher P50 levels than males across all age groups, with this trend being particularly pronounced in the young and longevity groups (Figure S1a). Further comparison analyses according to sex by age groups revealed no significant differences in P50 between males and females (Figure S1a). Thus, we demonstrated that the erythrocytes of longevity individuals had a more youthful oxygen release capacity than the elderly (the E Group).

Given that increased P50 is frequently considered a compensatory response to anemia and that the elderly are frequently facing anemia (Böning and Enciso 1987), we further determine whether the increased P50 of longevity individuals is a compensatory response to counteract anemia by assessing the erythrocyte parameters among all of the subjects. Figure 1b revealed a progressive decrease in RBC count, Hb, hematocrit (HCT), and mean corpuscular hemoglobin concentration (MCHC) with advancing age, while the mean corpuscular volume (MCV) demonstrated an increment with age. In the elderly group, RBC count and Hb levels as well as P50 were significantly decreased, indicating that compensatory elevation of P50 to combat anemia is decreased in the elderly. Notably, although RBC and Hb levels of longevity in individuals declined further compared to the elderly, they maintained the compensatory response to counteract anemia with a higher P50. Taken together, our findings indicate that anemia and decreased P50 are potential pathogenic features contributing to aging‐induced chronic tissue hypoxia. However, longevity individuals maintain compensatory mechanisms with efficient and youthful oxygen release capacity. This feature is a major previously unrecognized anti‐aging cellular system in longevity individuals to counteract age‐related anemia‐induced chronic peripheral tissue hypoxia.

Longevity individuals have a lower risk of inflammation (Monti et al. 2017; Ahuja et al. 2023). Thus, we further analyzed their inflammation status. The red cell distribution width with the coefficient of variation (RDWCV), is a hematological indicator that measures the variation in the size and volume of circulating erythrocytes and is frequently associated with inflammation, malnutrition, and impaired kidney function (Tian et al. 2023). We found that RDWCV was elevated in the elderly group and decreased in the longevity group relative to the elderly, implicating that the level of inflammation was lower in the longevity group compared to the elderly group. Another indicator reflecting the level of systemic inflammation, neutrophil–lymphocyte ratio (NLR), was also increased with age but not further increased in the longevity group. Thus, we confirmed that a lower level of inflammation was observed in the longevity group compared to the elderly group. Our findings immediately raise a new but compelling hypothesis that youth‐like oxygen release capability is an erythrocyte metabolomic feature associated with longevity individuals to combat age‐related anemia‐induced chronic peripheral tissue hypoxia, metabolic impairment, chronic inflammation, and tissue dysfunction.

To test the above hypothesis, we assessed the relationship among the erythrocyte oxygen‐release capability, organ function, and glycolipid metabolism indicators. Initially, we compared the age‐related clinical parameter outcomes among all of the participants. Specifically, although total serum protein (TPA) and albumin levels declined in longevity humans, they had lower alanine transaminase (ALT) levels, indicating better liver function. Indicators of renal function including creatinine, estimated glomerular filtration rate (eGFR), and urea nitrogen increased gradually with age, but plasma uric acid (UA), a biomarker of oxidative stress, declined in the longevity group. Moreover, there was a progressive increase in serum glucose levels through the aging process, but in longevity humans, blood glucose levels declined. A reduction in the harmful low‐density lipoprotein cholesterol (LDL) concentrations and the elevation of beneficial high‐density lipoprotein cholesterol (HDL) levels in longevity individuals may be interpreted as much better lipid metabolism. Thus, these results indicate that longevity is characterized by lower inflammation and better glucose‐lipid metabolism as well as indicators of better liver and kidney function (Figure 1b, Figure S1). Next, we analyzed the correlation between P50 and multiple clinical parameters (Figure 1c). Our findings revealed that the parameters associated with P50 varied across different age groups. In younger individuals, P50 showed a negative correlation with Hb, HCT, MCV, MCH, AGR, TBA, and systolic blood pressure (SBP), and a positive correlation with RDWCV, GLOB, UA, and creatinine. As age increased, the negative correlation between P50 and Hb concentration gradually decreased. In middle‐aged individuals, P50 showed a negative association with Hb, HCT, and creatinine, and a positive association with eGFR. In the elderly group, P50 showed a significant positive correlation with the inflammatory indicators, RDWCV, and NLR, but a negative correlation with HCT, WBC, lymphocyte count, ALB, and GLOB. In the longevity group, the correlation between P50 and RBC count and Hb concentration disappeared, only showing a negative correlation with MCV and a positive correlation with RDWCV and UA levels. UA may mirror not only the functionality of the kidneys but also the body's oxidative stress metabolism. Taken together, our findings strongly support our conclusion that the rejuvenation of oxygen release capacity is a newly identified longevity feature with better anti‐hypoxia, anti‐metabolic impairment, anti‐inflammation, and anti‐tissue damage capabilities, thereby better aging and longer lifespan.

### 2.3 Erythrocyte Metabolomic Signatures Associated With Oxygen Release, Anti‐Inflammation, and Oxidative Stress in Longevity Individuals

**FIGURE 2:**
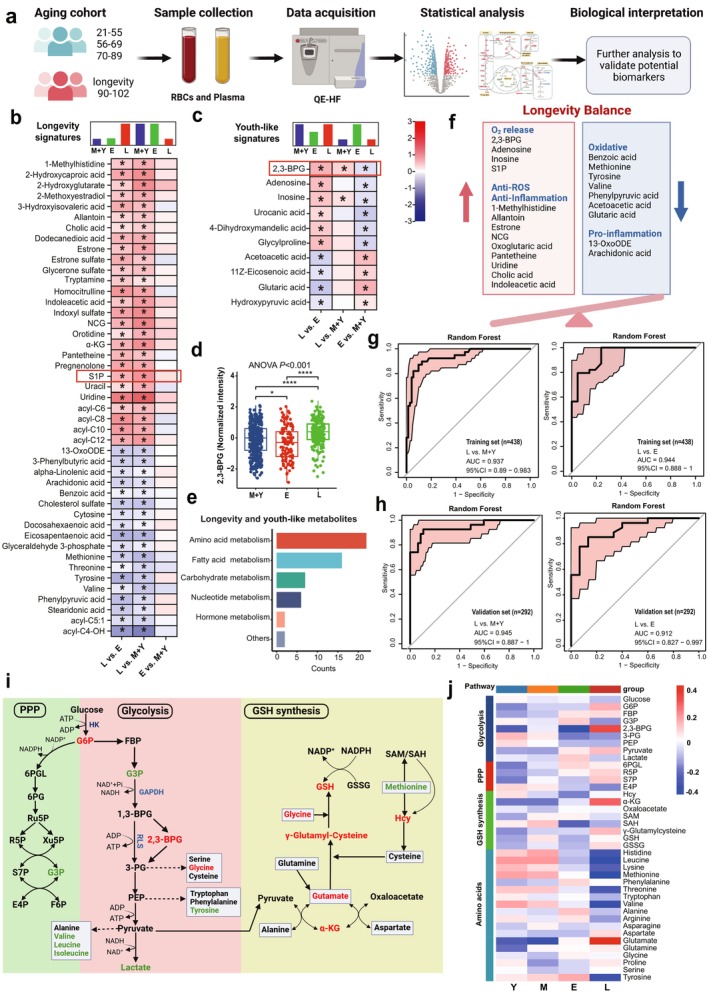
Erythrocyte metabolomics characteristics associated with oxygen release, anti‐inflammation, and oxidative stress in longevity individuals. (a) Schematic representation of the metabolomic study. (b, c) Changes in the relative abundance of erythrocyte metabolites between the longevity, elderly, middle‐aged, and young groups according to the following signatures of differential abundance: (1) longevity signatures and (2) youth‐like signatures. Each signature is accompanied by models depicting relative abundance patterns in longevity (L), elderly (E), and young and middle‐aged (M + Y) groups that would fall into the given signature. Color scale represents the fold changes (FC) between different groups: Longevity compared to elderly, longevity compared to young and middle‐aged, and elderly compared to young and middle‐aged. In each case, the latter group is used as a reference in the model. Red represents log_2_FC > 0 and blue represents log_2_FC < 0, *p* < 0.05 are indicated with asterisks. The longevity‐specific signatures are distinguished by a significant difference in metabolite abundance between longevity individuals and both the elderly and younger control groups while showing no significant variation within these control groups (b). The youth‐like signatures are characterized by metabolite levels that are comparable between longevity individuals and younger control participants (both young and middle‐aged) but differ significantly from those of elderly participants (c). 2,3‐BPG, 2,3‐bisphosphoglycerate; NCG, N‐carbamylglutamate; S1P, sphingosine‐1‐phosphate; α‐KG, oxoglutaric acid. (d) The normalized intensity of 2,3‐BPG in M + Y, E, and L group. Data are mean ± s.d. ****p < 0.0001, ***p < 0.001, **p < 0.01, *p < 0.05; one‐way ANOVA with Tukey's test. (e) The categories of longevity and youth‐like metabolites. Most metabolites are amino acids and their derivatives. (f) Summary of the longevity and youth‐like signatures of erythrocyte metabolites of longevity Individuals. (g) Receiver operating characteristics (ROC) curve for the random forest (RF) machine learning model based on the erythrocyte longevity and youth‐like signatures in training datasets. The plot represents the sensitivity (true positive rate) and the specificity (false positive rate) of the model. The area under the ROC curve (AUC) represents the entire area underneath the ROC curve and the confidence intervals (95% CI) are indicated in light red. The AUCs were 0.937 and 0.944 for the L versus M + Y group and the L versus E group. (h) ROC for RF model in erythrocyte validation datasets. The AUCs were 0.945 and 0.912 for the L versus M + Y group and the L versus E group. (i) Summary of the changes in erythrocyte glycolysis, the pentose phosphate pathway (PPP), and the glutathione (GSH) synthesis pathway. In specific pathways, red indicates metabolites that are elevated in the longevity group versus the elderly group, and green signifies reduced levels, with differences being statistically significant. 1,3‐BPG, 1,3‐bisphosphoglycerate; 3‐PG, 3‐phosphoglycerate; 6PG, 6‐Phosphogluconate; 6PGL, 6 phosphogluconolactonase; E4P, erythrose 4‐phosphate; F6P, fructose‐6‐phosphate; FBP, fructose bisphosphate; G3P, glyceraldehyde‐3‐phosphate; G6P, glucose‐6‐phosphate; GSSG, oxidized glutathione; Hcy, homocysteine; PEP, phosphoenolpyruvate; R5P, ribose 5‐phosphate; Ru5P, ribulose‐5‐phosphate; S7P, sedoheptulose‐7‐phosphate; SAH, s‐adenosylhomocysteine; SAM, s‐adenosylmethionine. (j) The heatmap of the metabolites of glycolysis, PPP, GSH synthesis, and amino acids in the longevity group (L), the elderly group (E), the middle‐aged group (M), and the young group (Y).

Erythrocytes have no nuclei, lack new protein synthesis, and their function is largely regulated by metabolic reprogramming (D'Alessandro et al. 2023). Thus, to determine how erythrocytes of longevity individuals maintain a youth‐like oxygen release capacity, we conducted high throughput untargeted metabolomic profiling to define the comprehensive metabolome of RBCs among all of the human subjects. The sample collection and processing, as well as the metabolomic analysis workflow, are shown in Figure 2a. Using high‐throughput LC–MS/MS methodology, we identified a total of 537 metabolites, with their main classifications depicted in Figure S2a. Due to the large sample size, we used the SERRF method for batch correction. The corrected QC samples clustered well, as shown in Figure S2b. The partial least squares discriminant analysis (PLSDA) showed that the erythrocyte metabolome of the longevity group could be differentiated from other groups, as illustrated in Figure S2c. Figure S3a–c presents the PLSDA plots from comparisons of groups, and the discrimination of the elderly and longevity group was obvious. The top 20 differential metabolites in different group comparisons are shown in Figure S3d–f (based on VIP in PLSDA). The pathway analysis of differential metabolites was performed using the Kyoto Encyclopedia of Genes and Genomes (KEGG) database (Figure S3g–i). The heatmap of all metabolites between groups showed varying metabolic trends among different groups. (Figure S3j). Venn diagram showed the comparison of the number of differential metabolites among all of the subjects during aging. There were the most differential metabolites between the longevity group and the elderly group (Figure S3k). These differential metabolites were enriched in 13 KEGG metabolic pathways (Figure S3l), including nucleotide metabolism such as purine and pyrimidine nucleotides, AA metabolism such as arginine, glycine, and glutamate, pentose phosphate pathway (PPP), and hormone metabolism (Figure S3l).

The current focus and challenge in longevity research is differentiating between aging biomarkers and longevity biomarkers. In this study, we categorized metabolites into three distinct age‐related signatures based on their trajectory with age, (1) the aging signature, characterized by the trajectory of metabolic changes closely correlated with age, either increasing or decreasing (Figure S2d,e); (2) the longevity‐specific signature, distinguished by a significant difference in metabolite abundance between longevity individuals and both the elderly and younger (both young and middle‐aged) control groups, but no significant difference between these two control groups (Figure 2b); and (3) the youth‐like signature, where metabolite levels are comparable between individuals of longevity and younger control participants (both young and middle‐aged), but differ significantly from the elderly participants (Figure 2c) (Sato et al. 2021). Notably, we found that longevity and youth‐like signatures were attributed mostly to AAs and their derived metabolites (Figure 2e). The longevity signatures were characterized by an enrichment of specific metabolites, such as 1‐methylhistidine, allantoin, cholic acid, estrone, N‐Carbamylglutamate (NCG), indoleacetic acid (IAA), oxoglutaric acid (α‐KG), pantetheine, and uridine. These metabolites are known for their antioxidative and anti‐inflammatory properties. Conversely, levels of 13‐oxoODE, arachidonic acid, benzoic acid, methionine, and tyrosine, which can induce oxidative stress and inflammation, were reduced in longevity individuals. Notably, 2,3‐bisphosphoglycerate (2,3‐BPG) (Figure 2d), adenosine, and inosine, which played crucial roles in regulating the erythrocyte oxygen release function, were included in the youth‐like signature and were enriched in longevity individuals. Furthermore, sphingosine 1‐phosphate (S1P), a vital erythrocyte function regulator, was a component of this longevity signature, exhibiting higher levels in longevity individuals. Thus, our metabolomics revealed the comprehensive metabolic signature of longevity and youth‐like in erythrocytes of longevity individuals, which is intimately associated with the youth‐like capability to deliver more oxygen, and better counteract oxidative stress and inflammation (Figure 2f).

We then conducted random forest (RF) networking training analyses to see how effectively those longevity and youth‐like signatures could differentiate longevity individuals from other age groups. Erythrocyte metabolomic datasets were split randomly into training and validation datasets with a ratio of 6:4. Receiver operating characteristics (ROC) curve for the RF machine learning model based on the erythrocyte longevity and youth‐like signatures in training and validation datasets (Figure S2f). The results presented in Figure 2g demonstrate that the erythrocyte longevity and youth‐like signatures exhibit strong discriminatory power in distinguishing between longevity individuals and controls. Specifically, the areas under the curve (AUCs) were calculated to be 0.937 and 0.944 for the training cohort comparisons of the L group versus the M + Y group and the L group versus the E group, respectively. Next, the classifiers were validated using the validation cohort. In this cohort, the erythrocyte longevity and youth‐like effectively differentiated longevity individuals from controls, achieving AUCs of 0.945 and 0.912, respectively (Figure 2h). These findings suggest that the longevity and youth‐like metabolites present in erythrocytes could potentially be utilized as a unique identifier to accurately distinguish longevity, demonstrating both a high level of sensitivity and specificity.

### 2.4 Enhanced Erythrocyte Rapoport–Luebering Shunt and GSH Synthesis of Longevity Individuals

To maintain the O_2_ delivery capacity and counteract the challenge of high reactive oxygen species (ROS) within RBCs, the metabolism of glucose as the major fuel for erythrocytes between glycolysis for energy need and the PPP coupling with the glutathione (GSH) synthesis for anti‐ROS is finely regulated under hypoxia. In particular, erythrocytes are highly enriched with Rapoport–Luebering Shunt (RLS) in the glycolytic pathway to generate 2,3‐BPG, as a potent negative allosteric modulator which lowers the hemoglobin and O_2_ binding affinity and in turn triggers more O_2_ delivery. Thus, to better understand the rejuvenating metabolic nature underlying youth‐like erythrocyte O_2_ delivery and anti‐oxidative stress capacity in longevity individuals, we closely examined glucose metabolic reprogramming among glycolysis‐RLS, PPP, and GSH as shown in Figure 2i,j. Specifically, in the glycolytic pathway (Figure 2i), longevity individuals showed higher levels of glucose 6‐phosphate (G6P) compared to the elderly group. Notably, glyceraldehyde‐3‐phosphate (G3P) exhibited an upward trend in the elderly group relative to the middle‐aged group, while G3P decreased and its downstream metabolite, 2,3‐BPG (Figure 2d), was significantly elevated in the longevity group compared to the elderly group. Similar to G3P, the lactate level in the elderly group increased compared to the middle‐aged group, but decreased in the longevity group compared to the elderly group, indicating a possible shift from the glycolysis pathway toward RLS and thus the higher production of 2,3‐BPG than the elderly group. In the PPP, 6‐phosphogluconate (6PGL) was higher in the elderly and the longevity groups in comparison to the young group. Sedoheptulose‐7‐phosphate (S7P) was higher in the middle‐aged and the longevity groups compared to the young group. D‐Erythrose 4‐phosphate was lower in longevity compared to the young and middle‐aged group. Thus, the upregulation of PPP occurred in both aging and longevity and did not exhibit unique longevity characteristics. Taken together, we revealed that glucose metabolism is wired toward RLS versus glycolysis without significant impact on PPP in erythrocytes of longevity individuals, leading to the higher production of 2,3‐BPG than the elderly group. The longevity group maintains a similar level of 2,3‐BPG as young and middle‐aged groups and thus its youth‐like O_2_ delivery capacity.

Next, we analyzed the GSH synthesis pathway as shown in Figure 2i,j. Intriguingly, GSH, γ‐glutamyl‐cysteine, and glycine levels were increased in the longevity group compared to the elderly group, while GSH levels were decreased in the elderly group compared to the middle‐aged group. Furthermore, glutamate, involved in the synthesis of GSH, was increased with age and reached the highest level in the longevity group. α‐KG, which is the glutamate source for GSH synthesis in human erythrocytes, was increased in the longevity group. Homocysteine decreased in the elderly group and increased in the longevity group. S‐adenosylhomocysteine (SAH) plays an important role in repairing damaged proteins, and it decreased in the elderly group and showed an increased trend in the longevity group. Methionine, an essential AA involved in the metabolism of polyamines and GSH, was decreased in the longevity group compared to the elderly group. Thus, erythrocytes in longevity individuals are characterized by an increased ability to synthesize GSH to combat oxidative stress.

Furthermore, the longevity group was equally divided into two subgroups based on the top and bottom P50 values, and their erythrocyte metabolic differences were analyzed. As depicted in Figure S2g,h, there were significant metabolic profile differences between these subgroups. Unexpectedly, metabolites typically associated with erythrocyte O_2_ release, such as 2,3‐BPG and adenosine, were absent among the differentially expressed metabolites. Instead, the differential metabolites were predominantly linked to antioxidant properties, including gentisic acid and allantoin. This finding suggests that metabolites related to O_2_ release may exhibit common characteristics among individuals with longevity, while within this population, P50 regulation appears to be independent of 2,3‐BPG and similar metabolites. It is likely that there are alternative metabolic pathways involved in the further regulation of youth‐like erythrocyte function within the longevity group.

### 2.5 Plasma Metabolomics of Longevity Individuals Demonstrated a Decrease in Hypoxia and an Increase in Aerobic Metabolism

**FIGURE 3:**
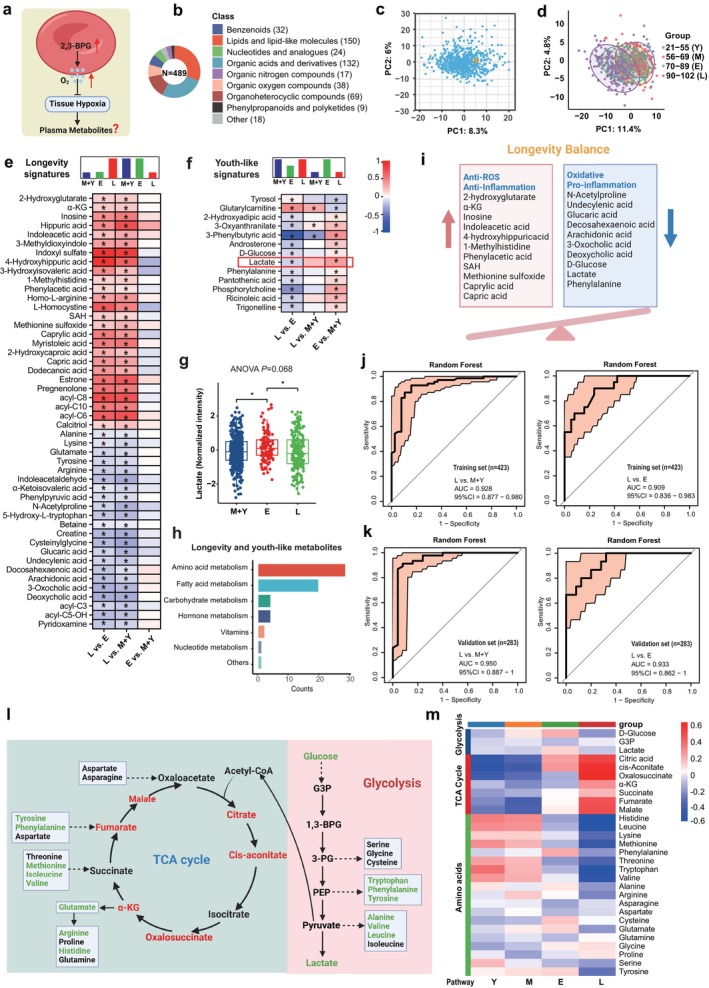
Plasma metabolomics showed a decrease in systemic hypoxia levels and an increase in aerobic metabolism in longevity individuals. (a) Schematic representation of the metabolomic study of plasma. (b) Chemical composition of plasma metabolites using the superclass from the Human Metabolome Database (HMDB) database. (c) Principal component analysis (PCA) of the plasma metabolome of 706 participants and corresponding quality control samples (QC, labeled orange), corrected QC samples clustered well, SERRF correction method for batch correction. (d) The partial least squares discriminant analysis (PLSDA) plot between different age groups. (e, f) Longevity and youth‐like plasma metabolites. Color scale represents the log_2_FC between different groups: longevity compared to the elderly, longevity compared to young and middle age, and elderly compared to young and middle age; in each case, the latter group is used as a reference in the model, red represents log_2_FC > 0, blue represents log_2_FC < 0, *p* < 0.05 are indicated with asterisks. (g) Boxplot of the normalized intensity of plasma lactate in M + Y, E, and L group. Data are mean ± s.d. ****p < 0.0001, ***p < 0.001, **p < 0.01, *p < 0.05; one‐way ANOVA with Tukey's test. (h) The categories of plasma longevity and youth‐like metabolites. (i) Representative metabolites associated with inflammation, oxidative stress, and hypoxia. (j) ROC curve for the RF machine learning model based on the plasma longevity and youth‐like signatures in training datasets. The plot represents the sensitivity (true positive rate) and the specificity (false positive rate) of the model. The AUC represents the entire area underneath the ROC curve and the confidence intervals (95% CI) are indicated in light red. The AUCs were 0.928 and 0.909 for the L versus M + Y group and the L versus E group. (k) ROC for RF model in plasma validation datasets. The AUCs were 0.950 and 0.933 for the L versus M + Y group and the L versus E group. (l) Summary of the changes of plasma glycolysis, tricarboxylic acid cycle (TCA) cycle pathway, and related amino acids. In specific pathways, red indicates metabolites are elevated in the longevity group versus the elderly group and green signifies reduced levels, with differences being statistically significant. (m) Heatmap of the plasma metabolites in glycolysis, TCA cycle pathway, and amino acids in age groups. Color indicates the magnitude of relative expression levels of different metabolites.

To determine if enhancement in the erythrocyte oxygen‐releasing capacity makes the longevity individuals have reduced systemic hypoxia, oxidative stress, and inflammation but better energy homeostasis, we employed untargeted metabolomics among all age groups and longevity individuals to investigate changes in plasma, which is often considered as an integrative readout of hypoxia and metabolic status of peripheral tissues (Figure 3a). We identified 489 plasma metabolites in total, with their primary classifications presented in Figure 3b. SERRF correction for batch correction is shown in Figure 3c, and the corrected QC samples clustered well. PLSDA analysis revealed distinct plasma metabolomic profiles among longevity and the other three age groups (Figure 3d). Figure S4a–c presents the PLSDA plots of the aging groups, and the segregation of the elderly and longevity groups was obvious. The top 20 differential metabolites in different group comparisons are shown in Figure S4d–f (based on VIP in PLSDA). The pathway analysis of differential metabolites was performed using the KEGG database (Figure S4g–i). The heatmap of all metabolites between groups showed varying metabolic trends among different groups (Figure S4j). Venn diagram showed the most differential metabolites between the longevity group and the elderly group (Figure S4k). Differential metabolites based on ANOVA analysis were enriched in 14 KEGG metabolic pathways (Figure S4l), such as arginine biosynthesis, valine, leucine and isoleucine biosynthesis, alanine, aspartate and glutamate metabolism, TCA (tricarboxylic acid) cycle, and cysteine and methionine metabolism.

Like erythrocytes, we categorized plasma metabolites into longevity and youth‐like signatures based on the trajectory with age (Figure 3e,f). An enrichment of anti‐ROS/inflammation metabolites was observed in the longevity signatures, such as α‐KG, indoleacetic acid, 1‐methylhistidine, caprylic acid, and capric acid. In contrast, representative hypoxia and inflammation‐related metabolites like arachidonic acid, 3‐oxocholic acid, and lactate were decreased in longevity humans. Notably, plasma glucose levels gradually increased from young to elderly and decreased in the longevity group, consistent with clinical data analysis (Table 1). The end product of the glycolytic pathway, lactate, was elevated in the elderly group but decreased in the longevity group, indicating a lower degree of tissue hypoxia in longevity individuals (Figure 3g). The longevity and youth‐like signatures mostly involved AA metabolism and fatty acid metabolism (Figure 3h). In general, similar to the metabolic characteristics of erythrocytes, we found that the longevity group exhibited an increase in antioxidative and anti‐inflammatory metabolites, and a decrease in pro‐oxidative stress and pro‐inflammatory metabolites in plasma as listed in Figure 3i.

The ROC for the RF model (Figure S5a) utilizing plasma longevity and youth‐like signatures also demonstrated strong performance in both the training and validation datasets. Specifically, the AUC values were 0.928 and 0.909 for distinguishing between the L versus M + Y group and L versus E group in the training cohort (Figure 3j), and 0.950 and 0.933 in the validation cohort (Figure 3k).

Consistent with longevity and youth‐like signatures, pathway enrichment among all of the differential metabolites between longevity and the elderly group revealed that most differential metabolites were closely related to AA metabolism and energy metabolism (Figure S4i). Figure 3l,m illustrates the changes in glycolysis, TCA cycle, and related AAs. Overall, longevity humans exhibited a decrease in the glycolysis pathway and an enhancement in the TCA cycle pathway. The levels of most essential AAs decreased in both the aging and longevity groups, with a more pronounced reduction in the longevity group, indicating their reduction is aging but not longevity signatures, while glycine, which is involved in GSH synthesis, was increased in the longevity group, indicating it is a potential longevity metabolite. Further quantitative analysis is required to verify these changes in AAs.

Similarly, we compared the plasma metabolic differences between the two subgroups of the longevity group with the highest and lowest 50 P50 values. We found that previously identified longevity factors such as S1P and IAA were significantly elevated in the high P50 group, suggesting that these metabolites may represent additional potential regulators of longevity, independent of oxygen release regulation (Figure S5b,c).

### 2.6 Youth‐Like Erythrocytes in Longevity Individuals Are Characterized by Higher Concentrations of S1P and Amino Acids Involved in GSH Synthesis

**FIGURE 4:**
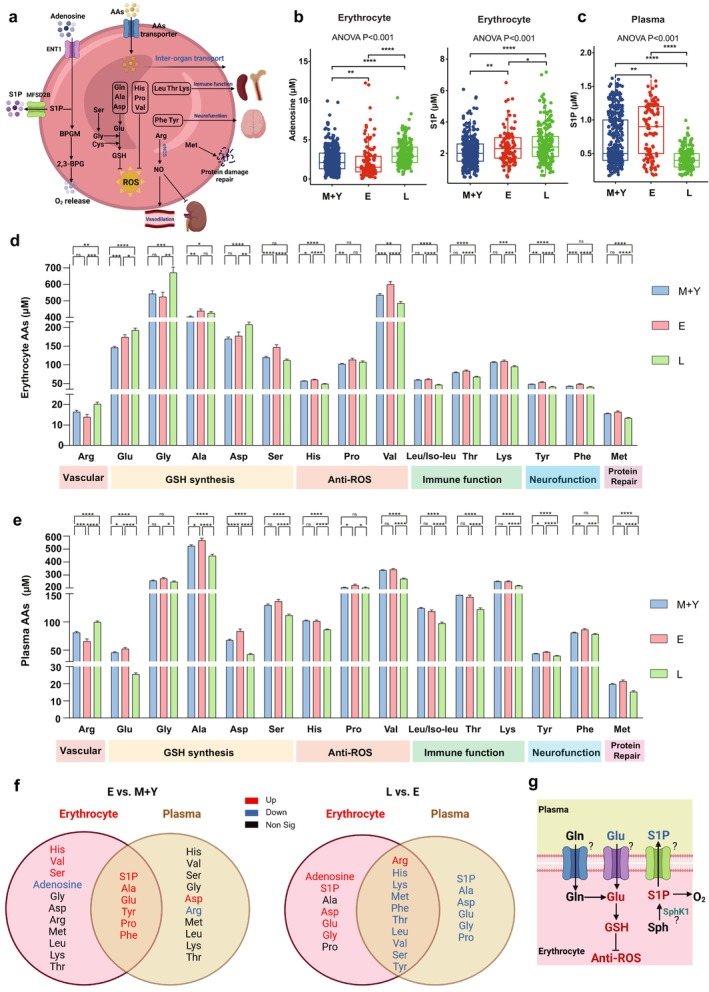
Quantitative of erythrocyte hypoxia sensors and amino acids and the crosstalk between erythrocyte and plasma. (a) The significant functions of adenosine and sphingosine‐1‐phosphate (S1P) in the regulation of oxygen release, as well as the involvement of amino acids (AAs) in erythrocyte glutathione (GSH) synthesis and anti‐reactive oxygen species (ROS) mechanisms, and their role in inter‐organ transport. (b, c) The specific erythrocyte concentration of adenosine and S1P, and plasma levels of S1P based on isotopic internal standards of all study participants. (d, e) The specific plasma concentration of amino acids based on isotopic internal standards of all study participants. Ala, alanine; Arg, arginine; Asp, aspartate; Glu, glutamate; Gly, glycine; His, histidine; Leu, leucine; Lys, lysine; Met, methionine; Phe, phenylanine; Pro, proline; Ser, serine; Thr, threonine; Tyr, tyrosine; Val, valine. Data are mean ± SEM. ****p < 0.0001, ***p < 0.001, **p < 0.01, *p < 0.05; Kruskal–Wallis with Dunn's test; ns, not significant. (f) Venn diagram of erythrocyte and plasma quantitative metabolites in aging (E vs. M + Y) and longevity (L vs. E) process. (g) Hypothesis that the crosstalk of S1P and glutamate between plasma and erythrocyte may contribute to mechanisms of longevity.

The results presented in the preceding section indicate that most youth‐like and longevity metabolites in erythrocytes and plasma were enriched in hypoxia sensors including S1P and adenosine, as well as AA metabolic pathways. The critical roles of S1P and adenosine in regulating oxygen release capacity have been well established in our published work (D'Alessandro and Xia 2020). Additionally, AAs play a crucial role in various physiological processes. They are involved in the synthesis of GSH within erythrocytes, potentially exhibit direct antioxidant activity against ROS, regulate vascular function, participate in the repair of protein damage, and contribute to the modulation of immune and neural functions through inter‐organ transport (Figure 4a). Thus, we further quantified the concentration of erythrocyte adenosine, erythrocyte and plasma S1P, and AAs of all study participants. During aging, erythrocyte adenosine showed a consistent rejuvenation pattern as seen with untargeted metabolomics; decreased in elderly adults when compared to the middle‐aged and young groups, while increased in longevity individuals (Figure 4b). Levels of erythrocyte S1P increased with age, peaking in the longevity group; plasma S1P increased in the elderly group, while in the longevity group it declined (Figure 4c). Figure 4d shows that levels of arginine (Arg), which is involved in NO synthesis, glutamate (Glu), aspartate (Asp), as well as glycine (Gly), which is involved in GSH synthesis, are significantly increased in the longevity group. Conversely, the levels of other AAs, except for proline (Pro), decreased in the longevity group. Next, quantitative analysis of AAs in plasma (Figure 4e) revealed a significant decrease in arginine levels with aging but reversed this trend to reach the highest levels in the longevity group, consistent with the changes observed in erythrocytes. The levels of other AAs in plasma decreased in the longevity group.

Erythrocytes have intimate communication with plasma by uptake or release of AAs or S1P to maintain erythrocyte and peripheral tissue biological function (Nemkov et al. 2018). Thus, to address the crosstalk between erythrocytes and plasma in terms of S1P and AAs, we compared the age‐dependent trajectory of all of these quantified metabolites between erythrocytes and plasma. As shown in Figure 4f during the aging process (elderly vs. middle‐aged and young adults), the metabolites that change similarly in erythrocytes and plasma were S1P, Ala, Glu, Tyr, Pro, and Phe, while those with different trends were His, Val, Ser, Asp, and Arg. During the longevity process (longevity vs. elderly), the metabolites that showed similar trends in erythrocytes and plasma were Arg, His, Ly, Met, Phe, Thr, Leu, Val, Ser, and Tyr. In particular, plasma arginine showed the same change pattern as erythrocytes, decreased in elderly adults when compared to the middle‐aged group, while increased in longevity individuals, implicating that arginine metabolism is enhanced in longevity individuals. In the comparison between the longevity group and the elderly group, metabolites with opposite trends in erythrocyte and plasma were markedly increased, including S1P, Ala, Asp, Glu, Gly, and Pro. Notably, S1P exhibited a decrease in the plasma of the longevity group, which is opposite to the results of erythrocytes. Thus, our results implicate the S1P transported from erythrocytes to plasma is reduced to maintain its similar levels as a young and middle‐aged group, while the uptake of AAs involved in GSH synthesis (Glu, Gly, and Ala), a neurotransmitter (Asp), and anti‐vasoconstriction (Arg) from plasma into erythrocyte exhibited longevity characteristics. The quantitative results and the comparison of exchange between plasma and erythrocytes suggest that mechanisms related to the increased synthesis of GSH in erythrocytes and the elevation of erythrocyte S1P, followed by its decreased release into plasma, might be one of the mechanisms contributing to longevity (Figure 4g).

### 2.7 The Molecular Mechanism Underlying Erythrocyte Metabolic Reprogramming and Youth‐Like Erythrocyte Oxygen Delivery in Longevity Individuals

**FIGURE 5:**
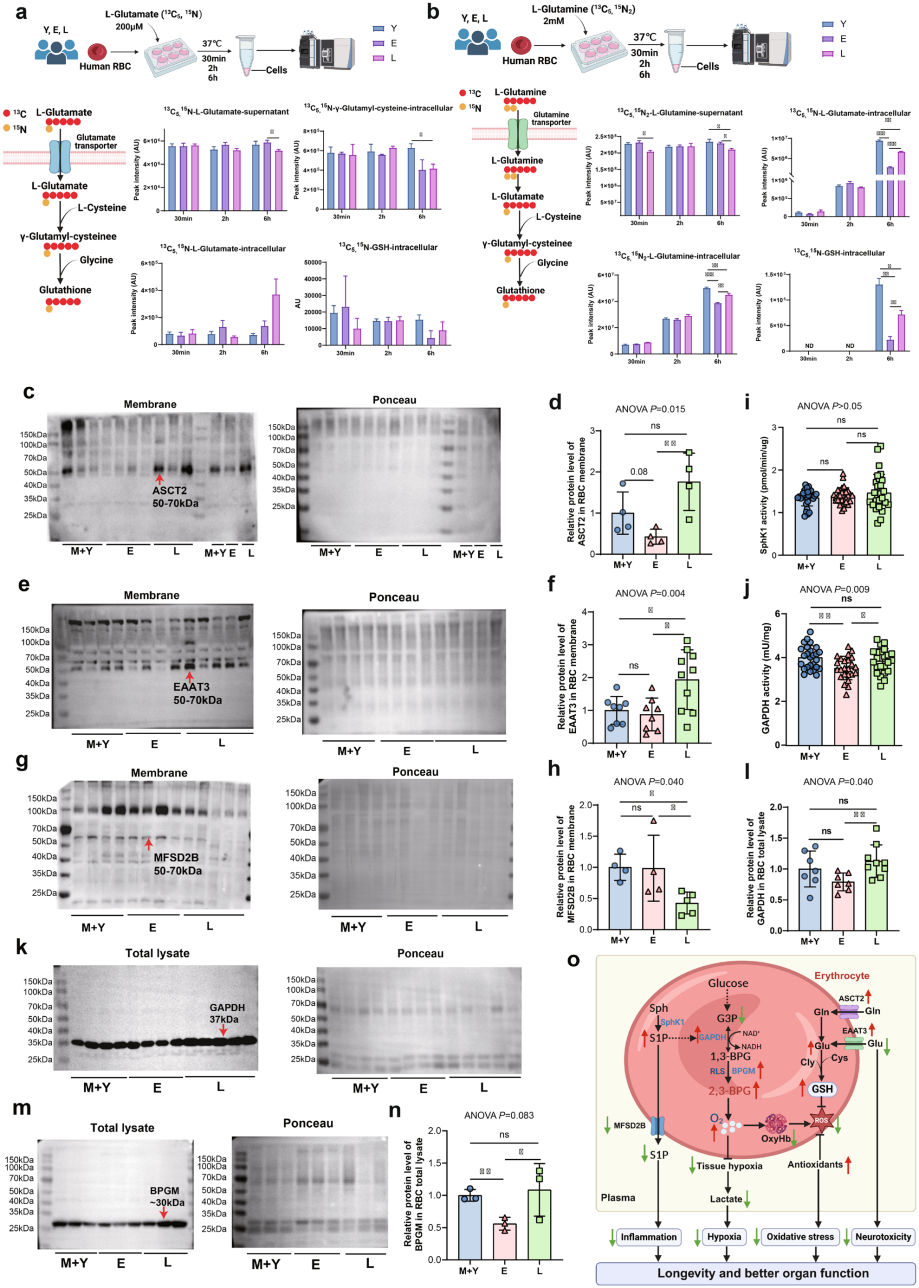
The mechanisms of enhanced erythrocyte GSH synthesis and maintained erythrocyte S1P In longevity individuals. (a) Isotopically labeled l‐glutamine flux experiments were conducted in cultured human RBCs to trace its metabolism in erythrocytes among young, elderly, and longevity individuals. (b) Isotopically labeled l‐glutamate flux experiments were conducted in cultured human RBCs to trace its metabolism in erythrocytes among young, elderly, and longevity individuals. (c) Protein expression of the alanine‐serine‐cysteine transporter type‐2 (ASCT2), a high‐affinity glutamine transporter, on RBC membrane (left) by western blot in three age groups, ponceau staining (right) as loading control. (d) The quantification of ASCT2 protein bands by densitometry, **p < 0.01, one‐way ANOVA. (e) Protein expression of the excitatory amino acid transporter 3 (EAAT3), transporter of glutamate and aspartate, on RBC membrane (left) by western blot in three age groups, ponceau staining (right) as loading control. (f) The quantification of EAAT3 protein bands by densitometry, *p < 0.05, one‐way ANOVA. (g) Protein expression of the major facilitator superfamily transporter 2b (MFSD2B), the exclusive S1P transporter, on RBC membrane (left) by western blot in three age groups, ponceau staining (right) as loading control. (h) The quantification of MFSD2B protein bands by densitometry, *p < 0.05, one‐way ANOVA. (i) Erythrocyte SphK1 activity in three age groups. ns, not significant, one‐way ANOVA. (j) Erythrocyte cytosolic GAPDH activity in three age groups, *p < 0.05, **p < 0.01, ns, not significant, one‐way ANOVA. (k) Protein expression of the GAPDH in RBC total lysate (left) by western blot in three age groups, ponceau staining (right) as loading control. (l) The quantification of GAPDH protein bands by densitometry, *p < 0.05, **p < 0.01, ns, not significant, one‐way ANOVA. (m) Protein expression of the BPGM in RBC total lysate (left) by western blot in three age groups, ponceau staining (right) as loading control. (n) The quantification of BPGM protein bands by densitometry, *p < 0.05, ns, not significant, one‐way ANOVA. (o) Working model: Longevity individuals exhibit a youth‐like erythrocyte oxygen release function, heightened anti‐ROS capacity, and reduced inflammation levels. This is accompanied by upregulation of the transporter responsible for glutamine and glutamate transport on the erythrocyte membrane in longevity individuals, resulting in elevated levels of glutamate and enhanced synthesis of glutathione. Furthermore, this mechanism contributes to the mitigation of neurotoxicity by decreasing plasma glutamate levels. The decreased membrane MFSD2B in RBC increases the intracellular level of S1P, which facilitates the release of membrane GAPDH, increased cytosolic GAPDH accelerates glycolysis and increases glycolytic RLS shunt (elevated 2,3‐BPG) which in turn leads to enhanced O_2_ delivery to counteract aging‐related tissue hypoxia and leading to longevity and healthy aging.

Intraerythrocytic glutamate can be derived from glutamine or by uptake from plasma. Glutamate enters erythrocytes via transporters, whereas human erythrocytes exhibit low basal permeability to glutamate and aspartate. However, under specific conditions, such as malaria infection, these excitatory AAs can enter erythrocytes against their concentration gradient via the excitatory amino acid transporter 3 (EAAT3) (Winterberg et al. 2012). Thus, to compare the uptake capability and intracellular metabolism of glutamine and glutamate by erythrocytes among young, elderly, and longevity groups, we isolated erythrocytes from young to longevity groups, followed by isotope‐flux tracing analyses in primary cultured erythrocytes. As illustrated in Figure 5a, we observed that the ^13^C_5_,^15^N‐labeled l‐glutamate in the supernatant exhibited a significant downward at 6 h in the longevity group compared to the elderly group. The intracellular ^13^C_5_,^15^N‐labeled l‐glutamate and ^13^C_5_,^15^N‐GSH levels in primary erythrocytes after being cultured for 6 h were relatively higher in the longevity group compared to the elderly group (Figure 5a). Similarly, at the 6 h incubation time point, the levels of ^13^C_5_,^15^N_2_‐labeled l‐glutamine in supernatant significantly decreased in the longevity group. The intracellular ^13^C_5_,^15^N_2_‐labeled l‐glutamine and its downstream metabolites including ^13^C_5_,^15^N‐glutamate and ^13^C_5_,^15^N‐GSH in cultured primary erythrocytes in the longevity group were significantly lower than the young group but higher than the elderly group. Moreover, the results of the western blot analysis indicate a significant increase in the protein expression of the alanine‐serine‐cysteine transporter type‐2 (ASCT2), a high‐affinity glutamine transporter, on the erythrocyte membrane in longevity individuals (Figure 5c,d). This trend was also observed in the membrane protein expression of EAAT3 (Figure 5e,f), indicating an enhanced, longevity‐specific transport of glutamine and glutamate from plasma to erythrocytes. Altogether, we demonstrated that increased l‐glutamine and l‐glutamate transporter expression levels coupled with enhanced intracellular metabolism lead to a higher production of GSH and higher anti‐oxidative stress capacity in the erythrocytes of longevity individuals.

To further explore the possible reason for the opposite trend of S1P in erythrocytes and plasma, we tested erythrocyte SphK1 activity in three age groups and found no significant difference between groups (Figure 5i). The increased erythrocyte S1P and decreased plasma S1P in longevity subjects may not be due to the elevated erythrocyte SphK1 activity. S1P is exported from erythrocyte to plasma, we assumed that the transporter, major facilitator superfamily transporter 2b (MFSD2B), may contribute to this longevity character. Supporting this possibility, we found that protein expression of the MFSD2B in the RBC membrane was significantly decreased in longevity (Figure 5g,h).

In our published work, S1P facilitates glycolysis under hypoxia by regulating the release of membrane‐anchored glycolytic enzymes such as GAPDH (Sun et al. 2016). Uniquely, our metabolomic data revealed that G3P and lactate levels were significantly reduced, while 2,3‐BPG levels were substantially induced in erythrocytes of longevity individuals, implicating that GAPDH and BPGM are likely two enzymes involved in channeling glucose metabolism toward RLS instead of glycolysis. Supporting this possibility, we found that the erythrocyte GAPDH activity was comparable between the young and middle‐aged group and longevity groups, both higher than that of the elderly group (Figure 5j). The protein level of GAPDH was highest in the longevity group, with a declining trend in the elderly group compared to the young and middle‐aged group (Figure 5k,l). Similarly, longevity humans maintained higher levels of bisphosphoglycerate mutase (BPGM) protein expression than the elderly (Figure 5m,n). Thus, we determined that longevity individuals maintain higher levels of both GAPDH and BPGM, which underlies their rejuvenated erythrocyte oxygen delivery capacity by promoting glucose metabolism switching to RLS versus glycolysis and inducing 2,3‐BPG generation.

In Figure 5o, we provide a comprehensive overview of the youth‐like and longevity signatures identified in the longevity process. Longevity individuals show a rejuvenation of erythrocyte oxygen release function, enhanced anti‐ROS capacity, and lower inflammation levels. This is accompanied by upregulation of the transporter responsible for glutamine and glutamate transport on the erythrocyte membrane in longevity individuals, resulting in elevated levels of glutamate and enhanced synthesis of GSH. Furthermore, this mechanism contributes to the mitigation of neurotoxicity by decreasing plasma glutamate levels. Notably, the decreased membrane MFSD2B in RBC led to increases in the intracellular level of S1P, which facilitates the release of membrane GAPDH, increased cytosolic GAPDH accelerates glycolysis and increases glycolytic RLS shunt (elevated 2,3‐BPG) which in turn leads to enhanced O_2_ delivery to counteract aging‐related tissue hypoxia and leading to longevity and healthy aging.

## 3 Discussion

Here, we defined the erythrocyte as a key cellular component with a previously unrecognized rejuvenation role in longevity featured with youth‐like metabolism and oxygen delivery capacity to better combat aging‐related tissue hypoxia, metabolic impairments, inflammation, and oxidative stress. In this way “youthful” erythrocytes help maintain better organ function and healthier aging. Overall, our findings have revealed a comprehensive youth‐like metabolic nature and function of erythrocytes in longevity and added a significant new chapter to our understanding of better aging in longevity and pave the way to promote healthy aging, prolong lifespan, and manage age‐related diseases.

Aging is a complex biological process involving multiple organs and cellular systems. For mammals, the absolute reliance on oxygen to generate ATP renders mammals extremely vulnerable to hypoxia. The aging‐related diseases are often accompanied by chronic tissue hypoxia, inflammation, and oxidative stress (Yeo 2019; Liu et al. 2023). Hypoxia has been long speculated as a driving force in the process of aging. Intriguingly, it is well known that longevity individuals are characterized by less hypoxia and more anti‐oxidative stress capability and better energy metabolism, with elevated fatty acid oxidation (FAO) (Li et al. 2022), high mtDNA copy members (He et al. 2016), and enhanced tricarboxylic acid cycle (TCA) intermediates (Mota‐Martorell et al. 2021). Mitochondrial TCA metabolites and antioxidants, such as pyruvate, fumarate, malate, α‐KG, oxaloacetate, and NAD^+^, have been previously shown to extend lifespan (Chin et al. 2014; Mouchiroud et al. 2011; Williams et al. 2009). However, why longevity individuals have a better energy metabolism and more antioxidants to cope with inflammation, oxidative stress metabolic impairment, and healthier aging (Yeo 2019; Liu et al. 2023) remains a mystery. Intriguingly, O_2_ is required to oxidize nutrients to produce sufficient energy and antioxidants to support normal life. For these reasons, oxygen is hailed as the “Elixir of Life”—a great tonic for medical therapies (Lane 2002). A large body of research has focused on the changes in erythrocyte number during aging and the role of such change in age‐related diseases (Yadav, Deepika, and Maurya 2024). For example, an early study reported a marked decrease in hemoglobin (Hb) levels and erythrocyte counts with advancing age (Takami et al. 2021). More studies indicate that multiple contributing factors including bone marrow failure and nutritional deficiencies underlie a decline in Hb levels in elderly (Stauder, Valent, and Theurl 2018). Further studies have highlighted low Hb levels as a potential risk factor for cognitive decline (Denny, Kuchibhatla, and Cohen 2006; Hong et al. 2013; Lucca et al. 2008). Similar studies pointed out that low Hb concentrations likely due to erythropoietin or iron deficiency potentially exacerbate oxidative stress and accelerate brain aging (Katsumi et al. 2021). However, functional changes of mature erythrocytes in healthy aging and longevity have not been reported prior to our present study. Here, we accurately measured O_2_ delivery capacity in a large human cohort showing that the erythrocyte O_2_ delivery capacity declined with increasing aging, while the longevity individuals have a youthful O_2_ release capacity and better anti‐ROS capability. Moreover, consistent with early findings, the longevity group in our cohort displays better aging features including lower levels of inflammation, better liver and kidney function, and improved glucose–lipid metabolism (Zhu et al. 2023b; Murata et al. 2023). Thus, in the elderly group, the decreased erythrocyte number (i.e., aging‐induced anemia) and decreased O_2_ release capacity are two potential pathogenic driving forces to initiate the aging‐induced chronic tissue hypoxia, thereby promoting glucose and lipid metabolic impairment, chronic inflammation, and progression to multiple tissue dysfunction. However, longevity individuals maintained compensatory responses with efficient and youth‐like oxygen release, which is a major previously unrecognized cellular defensive system to counteract age‐related anemia‐induced chronic peripheral tissue hypoxia, thereby less prone to develop metabolic impairment, inflammation, and tissue damage. Taken together, our findings immediately raise a new but compelling concept that age‐dependent progressive decline of erythrocyte counts (anemia) initiates chronic tissue hypoxia, while the youth‐like oxygen release capacity is a newly identified compensatory response for longevity individuals with better anti‐hypoxia (anemia), anti‐metabolic impairment, anti‐inflammation, and anti‐tissue damage capabilities, thereby promoting better aging and longer lifespan (see working model in Figure 5o). Such a youth‐like O_2_ release capacity could reflect better physiological factors, such as bone marrow and organ function reserves, lipid metabolism, and inflammation in longevity compared to the elderly.

Notably, erythrocytes are equipped with sophisticated metabolic regulatory machinery to sense hypoxia and release more oxygen by the reprogramming of glucose metabolism toward RLS to induce the synthesis of 2,3‐BPG. This process is activated by the adenosine A2B receptor (ADORA2B)‐S1P signaling cascade to mitigate hypoxia including high altitude (D'Alessandro and Xia 2020; Sun et al. 2016; Liu et al. 2016; Qiang et al. 2021; Zachara 1975), hemolytic disease (Sun et al. 2015), and chronic kidney disease (Peng et al. 2019; Xie et al. 2020). Moreover, early reports showed that erythrocyte 2,3‐BPG declines with age and is reduced in age‐matched individuals with Alzheimer's disease (Kosenko, Aliev, and Kaminsky 2016; Kaminsky et al. 2013). Intriguingly, recent mouse studies revealed that erythrocyte‐specific genetic ablation of *Adora2b* (Qiang et al. 2021) accelerates the early onset of age‐related hearing loss and cognitive decline (Qiang et al. 2021). However, to the best of our knowledge, the specific erythrocyte “youth‐like metabolites and molecules” for better oxygen delivery and better aging in longevity remain unknown. Our untargeted comprehensive metabolomic profiling highlighted that glucose metabolism is reprogrammed toward RLS versus glycolysis but has no significant impact on PPP in erythrocytes of longevity individuals, promoting higher 2,3‐BPG production than the elderly group to maintain youth‐like erythrocytes with rejuvenated O_2_ delivery capacity. To our surprise, untargeted metabolomics followed by the accurate quantification of S1P levels in both erythrocytes and plasma led us to confirm that the S1P, a key regulator of the erythrocyte hypoxic response is increased in the erythrocytes but reduced in the plasma due to the reduction of its specific transporter MFSD2B protein levels in the longevity group compared to the elderly. It is noteworthy that S1P is a bioactive lipid that can bind directly to Hb and promote deoxy‐Hb anchoring to the membrane, facilitating glycolysis under hypoxia by regulating the release of membrane‐anchored glycolytic enzymes such as GAPDH, thereby increasing glycolytic fluxes, 2,3‐BPG generation and ultimately increasing oxygen release (Sun et al. 2016). Supporting this finding, erythrocytes from the longevity individuals exhibit the increased activity of GAPDH, mirroring a rejuvenated characteristic similar to that of P50. Western blot analyses revealed that both GAPDH and BPGM protein levels are increased in the erythrocytes of the l longevity individuals compared to the elderly. Taken together, our studies led to a new working model that increased erythrocyte S1P due to decreased transporter MFSD2B protein levels coupled with increased GAPDH and BPGM protein levels work collaboratively as “erythrocyte youth‐like molecular machinery” to promote metabolic reprogramming with the induction of erythrocyte enriched “longevity metabolites” (S1P and 2,3‐BPG) and subsequent youthful oxygen delivery capacity.

Uniquely, the erythrocyte delivers oxygen but it does not use oxygen, Thus, erythrocytes constantly face substantial oxidative stress largely generated by auto‐oxidizing oxyhemoglobin (Fe^3+^HbO_2_) (Möller et al. 2023). To cope with such a high oxidative challenge, the erythrocyte is well poised to generate sufficient antioxidants such as NADPH from the PPP, which provides the reducing power necessary to synthesize sufficient amounts of the anti‐oxidant GSH (Orrico et al. 2023). A growing body of evidence indicates that individuals with exceptional longevity exhibit reduced oxidative damage with the lower level of plasma lipid peroxidation (Ngoi et al. 2021). Moreover, centenarians have significantly lower levels of oxidized proteins in plasma and reduced superoxide anion levels in neutrophils as well as significantly lower superoxide dismutase activity, higher GSH reductase activity, elevated levels of vitamins A and E, decreased levels of coenzyme Q10, and decreased susceptibility to lipid peroxidation compared to elderly controls (Belenguer‐Varea et al. 2020). Additionally, early studies revealed that erythrocyte antioxidant capacity decreases with age in mice with the reduction in GSH levels (Key et al. 2023). Furthermore, advancing age is accompanied by a chronic increase in basal systemic inflammation, termed inflammaging, contributing toward an increased risk of developing age‐related chronic diseases (Chambers and Akbar 2020; Dugan, Conway, and Duggal 2023). Excess oxygen levels may cause age‐related tissue damage and chronic inflammation by producing free radicals if the tissues cannot effectively use oxygen. Whether erythrocytes of longevity individuals maintain sufficient anti‐oxidants and whether the peripheral tissues are capable of effectively utilizing oxygen to generate sufficient energy and antioxidants to counteract age‐induced hypoxia, oxidative stress and inflammation are two remaining unresolved puzzles. Here, we demonstrated for the first time that erythrocytes of longevity individuals exhibit enhanced resistance to oxidative stress with an elevation of GSH and multiple GSH synthesis intermediates including glutamate and glycine compared to the elderly. Our findings highlight the significance of the erythrocyte dynamic between intracellular metabolism and transport functions across different age groups. Specifically, we revealed that erythrocytes from longevity individuals exhibit enhanced glutamate and glutamine transporter expression. As such, compared to the elderly, longevity individuals retain a greater capacity for the synthesis of GSH synthesis, a vital antioxidant that mitigates oxidative stress and protects cellular integrity during aging (Lapenna 2023). With higher GSH levels, erythrocytes in the longevity group have a higher and youth‐like capacity to counteract oxidative stress, thereby promoting the cellular health and combating cumulative damage associated with aging. Thus, increased glutamate and glutamine transporter protein expression coupled with enhanced intracellular metabolism are potential youth‐like molecular and metabolic bases responsible for better anti‐oxidative stress capacity in longevity. Correspondingly, the plasma metabolomic profiling revealed a reduction in the overall redox state as well as tissue hypoxia featured with enhanced TCA and decreased glycolytic intermediates in longevity individuals compared to the elderly group.

Chronic, low‐grade inflammation, often termed “inflammaging,” is a hallmark of aging. The metabolism of arachidonic acid and eicosanoids in erythrocytes stored in blood bank conditions has been shown to trigger pro‐immunogenic cascades and subsequent acute lung injury following transfusion (Nemkov et al. 2018). In our study, the inflammation homeostasis of longevity is reflected by the reduction of inflammation‐related metabolites and increase of anti‐inflammation metabolites in both erythrocytes and plasma in longevity individuals. Interestingly, the observed positive correlation between P50 and RDW‐CV, an established marker of inflammation, implies a potential interplay between oxygen release capacity and inflammatory status across different age groups (Y, M, and L groups). Elevated P50 values may signify an adaptive response to chronic inflammatory conditions, potentially enhancing oxygen release to satisfy the increased metabolic demands of inflamed tissues, thereby contributing to tissue oxygenation during inflammatory stress. Moreover, chronic inflammation is known to alter erythrocyte function and morphology, leading to increased RDW‐CV. The correlation between P50 and RDW‐CV suggests that inflammation may influence erythrocyte functionality, possibly modifying hemoglobin's oxygen affinity as a compensatory mechanism. This interplay underscores the complex relationship between oxygen delivery, inflammation, and erythrocyte dynamics in longevity. Altogether, our findings provide comprehensive erythroid and systemic metabolic snapshots featuring chronic hypoxia, metabolic impairment, excessive oxidative stress, and inflammation in the elderly but youth‐like metabolic features including less hypoxia, oxidative stress, and inflammation in the longevity group.

Erythrocytes have robust membrane transporters, making the mature erythrocyte a circulating reservoir for many essential nutrients, especially AAs (Nemkov et al. 2018). Humans have similar or higher levels of AAs in erythrocytes compared with their corresponding serum levels, and significant correlation coefficients showed that strong plasma‐erythrocyte relationships existed for essential AAs including alanine, valine, methionine, isoleucine, leucine, and phenylalanine (Agli et al. 1998). Notably, dietary restriction with essential AAs such as methionine and the branched‐chain AAs (isoleucine/leucine and valine) is helpful in delaying aging and extending healthspan and lifespan (Czibik et al. 2021). However, the specific changes and quantification of AAs during aging and longevity in erythrocytes and plasma remained undetermined. Our untargeted metabolomics profiling revealed that AAs are the top‐ranked youth‐like and longevity‐related metabolites among all of the identified metabolites. Further accurate quantification of all of the AAs revealed the decline of methionine, valine, and phenylalanine in both erythrocytes and plasma of longevity individuals compared to the elderly, supporting the “dietary restriction” theory (Czibik et al. 2021). Moreover, the crosstalk between erythrocytes and plasma also reflects an increased transport of certain AAs involved in GSH synthesis (glutamate, aspartate, and glycine) and arginine which participate in NO production, into erythrocytes during the longevity phase. Concurrently, S1P, primarily produced and released into the plasma by erythrocytes (Sun et al. 2016), shows a decreased transport from erythrocytes to plasma. A prominent feature of the longevity humans is an increase in erythrocyte intracellular S1P, coupled with a decrease in plasma S1P levels due to a notable decrease in MFSD2B protein expression on the erythrocyte membrane of longevity individuals. Taken together, our findings support an innovative concept that erythrocytes act as “migrating organs” sensing hypoxia and releasing more O_2_ to combat tissue hypoxia but also carrying and transporting metabolites to peripheral tissues to maintain nutrient exchange, redox homeostasis, immune cell responses, vasoactive balance, and even neurotransmitter release to support better and healthier aging.

In conclusion, our findings provide multiple new insights into the function and mechanisms underlying better aging and longevity, including the following features: (i) erythrocyte youth‐like function and rejuvenated metabolic nature in longevity individuals characterized by efficient oxygen release, robust anti‐hypoxia, anti‐oxidative stress, anti‐inflammation, and anti‐metabolic impairment and thus less organ dysfunction, better aging, and longevity and (ii) reduced MFSD2B leading to decreased S1P release to allow more intracellular S1P stored within erythrocytes along with increased GAPDH, BPGM, and glutamine/glutamate transporter protein levels working collaboratively as a newly identified rejuvenation defensive machinery to induce GAPDH activity, more 2,3‐BPG production, O_2_ delivery, and GSH production to counteract tissue hypoxia, oxidative stress, inflammation and metabolic impairment, and thus longevity. Significantly, our newly identified and quantified metabolites in both erythrocytes and plasma have the potential to predict longevity. Finally, given the global increase in average lifespan and the consequent rise in age‐related diseases, our study further raises the possibility of using the erythrocyte as a biomarker for health monitoring and as a therapeutic target for extending lifespan. In the future, validating our findings in independent cohorts, conducting long‐term follow‐up studies in elderly populations, and undertaking more in‐depth mechanistic investigations to explore the impact of the identified longevity and youth‐like metabolites on aging and longevity are all promising avenues for further research.

## 4 Experimental Procedures

### 4.1 Ethics Approval and Consent to Participate

This study was approved by the Ethics Committee of Xiangya Hospital (202209002 and 202310207). All participants signed an informed consent agreement before donating their blood samples.

### 4.2 Participant Information and Sample Collection

All participants (21–102 years old) were living within the community in Hunan province, China. Detailed health‐related data were obtained using a face‐to‐face questionnaire (Table S1) that covered living conditions, demographics information, clinical and medical history, dietary habits, drug usage, and lifestyle. In addition, essential health metrics including height, weight, and blood pressure were collected or assessed by the researchers. Individuals over the age of 90 were classified as longevity people based on criteria used in prior research (Escourrou et al. 2022; Fernández‐Blázquez et al. 2021; Franceschi et al. 2007). Exclusion criteria for participant enrollment were history of or current diagnosis of malignant tumors; history of immunodeficiency, or suffering from other acquired or congenital immunodeficiency diseases; severe infection, liver or kidney dysfunction; and hematologic disorders or history of blood transfusion (see flowchart in Figure S6). The body mass index (BMI) was calculated by dividing body weight in kilograms by the square of height in meters. Laboratory parameters included complete blood count, liver and kidney function tests, and measurements of blood glucose and lipid levels. These tests were conducted in accredited local medical institutions, ensuring the accuracy and reliability of the results. eGFR of the human subjects was calculated based on the Modification of Diet in Renal Disease (MDRD) equation: eGFR = 186 × serum creatinine^−1.154^ × age^−0.203^ × 0.742 (if female). Detailed information on the enrolled participants is presented in Table 1.

All participants were fasted for more than 8 h. Blood samples were collected by venous puncture into EDTA‐anticoagulation tubes and then centrifuged at 2000 *g* for 5 min at 4°C. Post‐centrifugation, the plasma (upper layer) and the erythrocytes (lower layer) were carefully separated. These samples were then rapidly frozen using liquid nitrogen and subsequently placed at −80°C for long‐term storage. The total number of erythrocyte samples is 730, while the number of plasma samples is 706, as there were 24 plasma samples did not pass metabolic quality control.

### 4.3 Erythrocyte Oxygen Release Capacity (P50) Measurement

The partial pressure of O_2_ required for 50% Hb binding to O_2_, known as P50, was determined as follows (Xie et al. 2020; Xu et al. 2022), 20 μL whole blood sample was combined with 5 mL Hemox Buffer (TCS Scientific Corporation), 10 μL anti‐foaming reagent (TCS Scientific Corporation), and 20 μL additive‐A (22% bovine serum albumin). This mixture was then analyzed using a Hemox Analyzer (TCS Scientific Corporation) to measure the oxygen equilibrium curve at 37°C. The P50 value was calculated using the OEC3 software.

### 4.4 Untargeted Erythrocyte and Plasma Metabolomic Analysis

Erythrocytes and plasma were diluted in lysis solution (methanol:acetonitrile:water 5:3:2 v/v/v) at ratios of 1:10 and 1:25, respectively. The suspensions were vortexed continuously for 30 min at 4°C and centrifuged at 18,213 *g* for 10 min at 4°C. Supernatants were then injected into an ultra‐high‐pressure liquid chromatography–MS (UHPLC–MS) using a Vanquish UHPLC coupled with a Q Exactive MS (Thermo Fisher, Bremen, Germany). Metabolite analysis was conducted using a 5‐min gradient, following previously described methods (Chen et al. 2023; Nemkov et al. 2019). Briefly, metabolites were separated on a Kinetex C18 column (150 × 2.1 mm, 1.7 μm, Phenomenex, 00F‐4475‐AN) under these conditions: flow rate of 0.45 mL/min, column temperature at 45°C, and sample compartment temperature at 7°C. The positive solvent gradient was: 0–0.5 min at 5% B, 0.5–1.1 min from 5% to 95% B, 1.1–2.75 min holding at 95% B, 2.75–3 min from 95% to 5% B, and 3–5 min holding at 5% B (A: 0.1% formic acid in water, B: 0.1% formic acid in acetonitrile). The negative solvent gradient was: 0–0.5 min at 0% B, 0.5–1.1 min from 0% to 100% B, 1.1–2.75 min holding at 100% B, 2.75–3 min from 100% to 0% B, and 3–5 min holding at 0% B (A: 5% acetonitrile/95% water/1 mM ammonium acetate, B: 95% acetonitrile/5% water/1 mM ammonium acetate). Samples were randomized and analyzed in both positive and negative ion modes. The mass spectrometer operated in full MS mode at a resolution of 70,000, a scan range of 65–900 *m*/*z*, a maximum injection time of 200 ms, with 2 micro scans, and an automatic gain control of 3 × 10^6^ ions. Source voltage was set at 4.0 kV for both ion modes, with a capillary temperature of 320°C, and nitrogen was used for sheath gas (45), auxiliary gas (15), and sweep gas (0).

Raw data files were converted to mzXML format using RawConverter (Scripps Research Institute) and analyzed via Maven (Princeton University, Princeton, New Jersey, USA). Instrument stability and quality control were ensured through replicate injections of a technical mixture every 10 runs.

### 4.5 Targeted Quantification of Erythrocyte and Plasma Metabolites

In summary, 20 μL of red blood cells were placed into a 1.5 mL Eppendorf tube, and 180 μL of a pre‐chilled (−20°C) extraction solution containing stable isotope internal standards was added. The subsequent steps followed established procedures. Quantification relied on the integrated peak areas of extracted ion chromatograms at the MS1 level.

### 4.6 Isotopically Labeled l‐Glutamine and l‐Glutamate Flow Analyses in Cultured Human Erythrocytes of Different Age Groups

Three individuals were selected from each of the young, elderly, and longevity populations. Erythrocytes were isolated from blood with heparin as an anticoagulant for isotope flux experiments. The packed erythrocytes were then purified via Percoll density purification (Sigma‐Aldrich), following the method previously described (Xu et al. 2022; Chen et al. 2023). These purified erythrocytes were added to the F‐10 Nutrient Mix (Invitrogen) containing isotopes to achieve an HCT of 4%. The erythrocytes were added to each well of a 12‐well plate and cultured with 2 mM of ^13^C_5_,^15^N_2_‐labeled l‐glutamine under normoxia conditions at 37°C for 30 min, 2 and 6 h, as well as with 200 μM of ^13^C_5_,^15^N‐labeled l‐glutamate. Erythrocytes were collected and lysed as mentioned above and analyzed using a Vanquish UHPLC coupled to a Q Exactive MS (Thermo Fisher, Bremen, Germany). Metabolite identification and isotopologue distribution analysis were performed using Maven (Princeton, NJ).

### 4.7 Isolation of RBC Cytoplasm and Measurement of GAPDH Activity

RBCs were lysed by freeze–thaw cycles in 10 volumes of 5 mM cold phosphate buffer (pH 8.0) followed by vortexing. RBC membrane was removed by centrifuging at 20,000 *g* for 20 min at 4°C. The supernatant was saved and used to measure cytosolic GAPDH activity with a GAPDH assay kit (Abcam, # ab284542).

### 4.8 Sphk1 Activity Assay

Erythrocyte Sphk1 activity was assessed using methods previously detailed (Xie et al. 2020). In summary, RBCs were lysed in a solution containing 50 mM HEPES pH 7.4, 15 mM MgCl2, 0.05% Triton X‐100, 10 mM KCl, and protease inhibitors. Next, 100–300 μg of RBC lysate was incubated with 5 μM NBD‐Sphingosine (Avanti 810205) in 1% fatty acid‐free BSA within a total reaction volume of 100 μL (comprising 50 mM HEPES pH 7.4, 15 mM MgCl_2_, 0.05% Triton X‐100, 10 mM KCl, 10 mM NaF, 1.5 mM semicarbazide, and 1 mM ATP) at 37°C for 30 min. The resulting lipid NBD‐S1P was extracted using 100 μL of 1 M potassium phosphate buffer (pH 8.5) and 500 μL of chloroform/methanol (2:1), followed by centrifugation at 15,000 rpm for 1 min. Subsequently, 100 μL of the supernatant was transferred to a 96‐well fluorescence assay plate to measure fluorescence at 489/535 nm (excitation/emission) to quantify NBD‐S1P levels using NBD‐S1P standards.

### 4.9 Western Blot Analyses

Western blot analysis of total and membrane protein from human erythrocytes was conducted as previously described (Xu et al. 2022). Samples were loaded with 10% SDS–PAGE gel for Western blot analysis. We used primary antibodies to ASCT2 (Proteintech, # 20350‐1‐AP, 1:8000), EAAT3 (Proteintech, # 12686‐1‐AP, 1:1000), MFSD2B (Invitrogen, # PA5‐21050, 1:1000), GAPDH (Proteintech, # 60004‐1‐Ig, 1:3000), BPGM (Proteintech, # 17173‐1‐AP, 1:4000), and then incubated with secondary antibodies (Abiowell, # AWS0001), Goat anti‐Mouse IgG (H + L), HRP, 1:5000; # AWS0002, Goat anti‐Rabbit IgG (H + L), HRP; 1:5000. Ponceau S Stain of total proteins was used as loading controls (Beyotime).

### 4.10 Statistical Analysis

The metabolomic data were normalized based on the website MetaboAnalyst (https://www.metaboanalyst.ca/MetaboAnalyst/). The metabolite intensity values were initially log‐transformed and autoscaled (mean‐centered and normalized by the standard deviation (SD) of each variable). To evaluate group differences, PLSDA was employed. Metabolites with *p*‐values < 0.05 were identified as differential biomarkers. Metabolic pathway analysis was performed through the Pathway Analysis module in MetaboAnalyst 5.0, utilizing the KEGG pathway library (https://www.kegg.jp). Statistical tests were performed in R version 4.2.2 and GraphPad Prism 8.0. Continuous variables were reported as mean ± SD or median with interquartile range (IQR), based on their distribution (normal or non‐normal), while categorical variables were expressed as counts and percentages. Pearson's *χ* ^2^ test was employed for categorical data. For continuous data, the t‐test was applied for normally distributed variables, and the Mann–Whitney *U* tests for non‐normal distributions. One‐way ANOVA with Tukey's test (parametric) and Kruskal–Wallis with Dunn's test (nonparametric) were used for multiple comparisons. Spearman's correlations between P50, clinical variables, and metabolites were analyzed using GraphPad Prism 8.0, and the correlation heatmap was generated using the Hiplot tools (https://hiplot.com.cn/). The study population was randomly divided into training and validation datasets in a 6:4 ratio for machine learning analysis. The performance of the RF model was assessed using ROC curve analysis with the pROC package (v.4.1.0). The model's discriminative ability was quantified by the AUC. For all analyses, a *p* value < 0.05 was considered statistically significant.

## Author Contributions

F.Y., W.L., and C.C. designed and performed the data analysis. J.L., Z.Yang, and C.C. performed the metabolomics screening. C.C., Z.Z., Y.F., Z.L., and W.H. collected the blood samples and health information from each participant. T.X. and Y.Z. participated in the study design. C.C., Q.G., and Z.Yao performed P50 measurements. F.Y. wrote the manuscript. R.E.K. and J.X. provided expertise in proofreading the manuscript. C.L. provided advice on data analysis. J.L. and Y.X. designed and supervised the human subject. Y.X. oversaw the design of experiments, the interpretation of results, the writing and organization of the manuscript, and did the final editing. All authors approved the final version of the manuscript.

## Conflicts of Interest

The authors declare no conflicts of interest.

## Supporting information


**Figure S1.** Sexual difference of erythrocyte function in age groups and other clinical variables comparisons between groups. a, Comparison of P50 between females and males in different age groups. b, Boxplots for comparing clinical variables between groups. HCT, hematocrit; MCV, mean corpuscular volume; MCH, mean corpuscular hemoglobin; MCHC, mean corpuscular hemoglobin concentration; N, neutrophil; AST, aspartate aminotransferase; TPA, total serum protein; ALB, albumin; GLOB, globulin; AGR, albumin–globulin ratio; TBil, total bilirubin; DBil, direct bilirubin; TBA, total bile acid; eGFR, estimated glomerular filtration rate; TG, triglyceride; TC, total cholesterol; BMI, body mass index; SBP, systolic blood pressure; DBP, diastolic blood pressure. Data are mean ± s.d. ****p < 0.0001, ***p < 0.001, **p < 0.01, *p < 0.05; Kruskal–Wallis with Dunn’s test; ns, not significant.
**Figure S2.** Erythrocyte metabolomics analysis and the aging signatures. a, Chemical composition of erythrocyte metabolites using the super class from HMDB database. b, Principal component analysis (PCA) of the erythrocyte metabolome of 730 participants and corresponding Quality Control samples (QC, labeled orange). QC samples are highly clustered, demonstrating low technical variance and high metabolomic reliability, SERRF correction method for batch correction. c, The partial least squares discriminant analysis (PLSDA) plot between different age groups. d‐e, Changes in the relative abundance of erythrocyte metabolites between the longevity, elderly, middle‐aged, and young groups according to aging signatures of differential abundance. The aging signatures included taxa whose abundance was increased or decreased with age. f, Flowchart illustrating the construction of a machine learning model based on erythrocyte longevity and youth‐like metabolites. g, PLSDA plot showing the metabolic differences in erythrocytes between the high P50 and low P50 subgroups within the longevity group. h, Volcano plot depicting the metabolic differences in erythrocytes between the high P50 and low P50 subgroups within the longevity group.
**Figure S3.** Erythrocyte metabolomics analysis for all the samples. a, PLSDA plot showing metabolic features of Y (red) and M (green) groups. b, PLSDA plot showing metabolic features of M (red) and E (green) groups. c, PLSDA plot showing metabolic features of E (red) and L (green) groups. d‐f, VIP values and ranking of top 20 molecules according to PLSDA analysis of different age group comparisons. g–i, KEGG pathway enrichment analysis based on differentially abundant metabolites between groups. j, Overall heatmap of all metabolites. k, Venn diagram showing the overlap of differentially expressed erythrocyte metabolites between different groups. l, The KEGG pathway analysis of all differential metabolites between groups.
**Figure S4.** Plasma metabolomics analysis for all the samples. a, PLSDA plot showing metabolic features of Y (red) and M (green) groups. b, PLSDA plot showing metabolic features of M (red) and E (green) groups. c, PLSDA plot showing metabolic features of E (red) and L (green) groups. d‐f, VIP values and ranking of top 20 molecules according to PLSDA analysis of different age group comparisons. g–i, KEGG pathway enrichment analysis based on differentially abundant metabolites between groups. j, Overall heatmap of all metabolites. k, Venn diagram showing the overlap of differentially expressed plasma metabolites between different groups. l, The KEGG pathway analysis of all differential metabolites between groups.
**Figure S5.** Plasma metabolomics analysis for high P50 versus low P50 group within longevity individuals. a, Flowchart illustrating the construction of a machine learning model based on plasma longevity and youth‐like metabolites. b, PLSDA plot showing the metabolic differences in plasma between the high P50 and low P50 subgroups within the longevity group. c, Volcano plot depicting the metabolic differences in plasma between the high P50 and low P50 subgroups within the longevity group.
**Figure S6.** Flowchart of participant selection.
**Table S1.** Detailed questionnaire for assessing health conditions.

## Data Availability

Because of participant confidentiality and privacy concerns, data are available upon written request. All data are available from the corresponding author upon reasonable request.
