# Supplementary material for: Longevity Humans Have Youthful Erythrocyte Function and Metabolic Signatures
Source: Aging Cell. 2025 Feb 9;24(5):e14482. doi: 10.1111/acel.14482 (PMC12074018; doi:10.1111/acel.14482)
Supplement: Supplementary file 1 — Figure S1. Sexual difference of erythrocyte function in age groups and other clinical variables comparisons between groups. a, Comparison of P50 between females and males in different age groups. b, Boxplots for comparing clinical variables between groups. HCT, hematocrit; MCV, mean corpuscular volume; MCH, mean corpuscular hemoglobin; MCHC, mean corpuscular hemoglobin concentration; N, neutrophil; AST, aspartate aminotransferase; TPA, total serum protein; ALB, albumin; GLOB, globulin; AGR, albumin–globulin ratio; TBil, total bilirubin; DBil, direct bilirubin; TBA, total bile acid; eGFR, estimated glomerular filtration rate; TG, triglyceride; TC, total cholesterol; BMI, body mass index; SBP, systolic blood pressure; DBP, diastolic blood pressure. Data are mean ± s.d. ****p < 0.0001, ***p < 0.001, **p < 0.01, *p < 0.05; Kruskal–Wallis with Dunn’s test; ns, not significant. Figure S2. Erythrocyte metabolomics analysis and the aging signatures. a, Chemical composition of erythrocyte metabolites using the super class from HMDB database. b, Principal component analysis (PCA) of the erythrocyte metabolome of 730 participants and corresponding Quality Control samples (QC, labeled orange). QC samples are highly clustered, demonstrating low technical variance and high metabolomic reliability, SERRF correction method for batch correction. c, The partial least squares discriminant analysis (PLSDA) plot between different age groups. d‐e, Changes in the relative abundance of erythrocyte metabolites between the longevity, elderly, middle‐aged, and young groups according to aging signatures of differential abundance. The aging signatures included taxa whose abundance was increased or decreased with age. f, Flowchart illustrating the construction of a machine learning model based on erythrocyte longevity and youth‐like metabolites. g, PLSDA plot showing the metabolic differences in erythrocytes between the high P50 and low P50 subgroups within the longevity group. h, Volcano plot d [file ACEL-24-e14482-s001.zip › Supplementary materials revised.pdf]

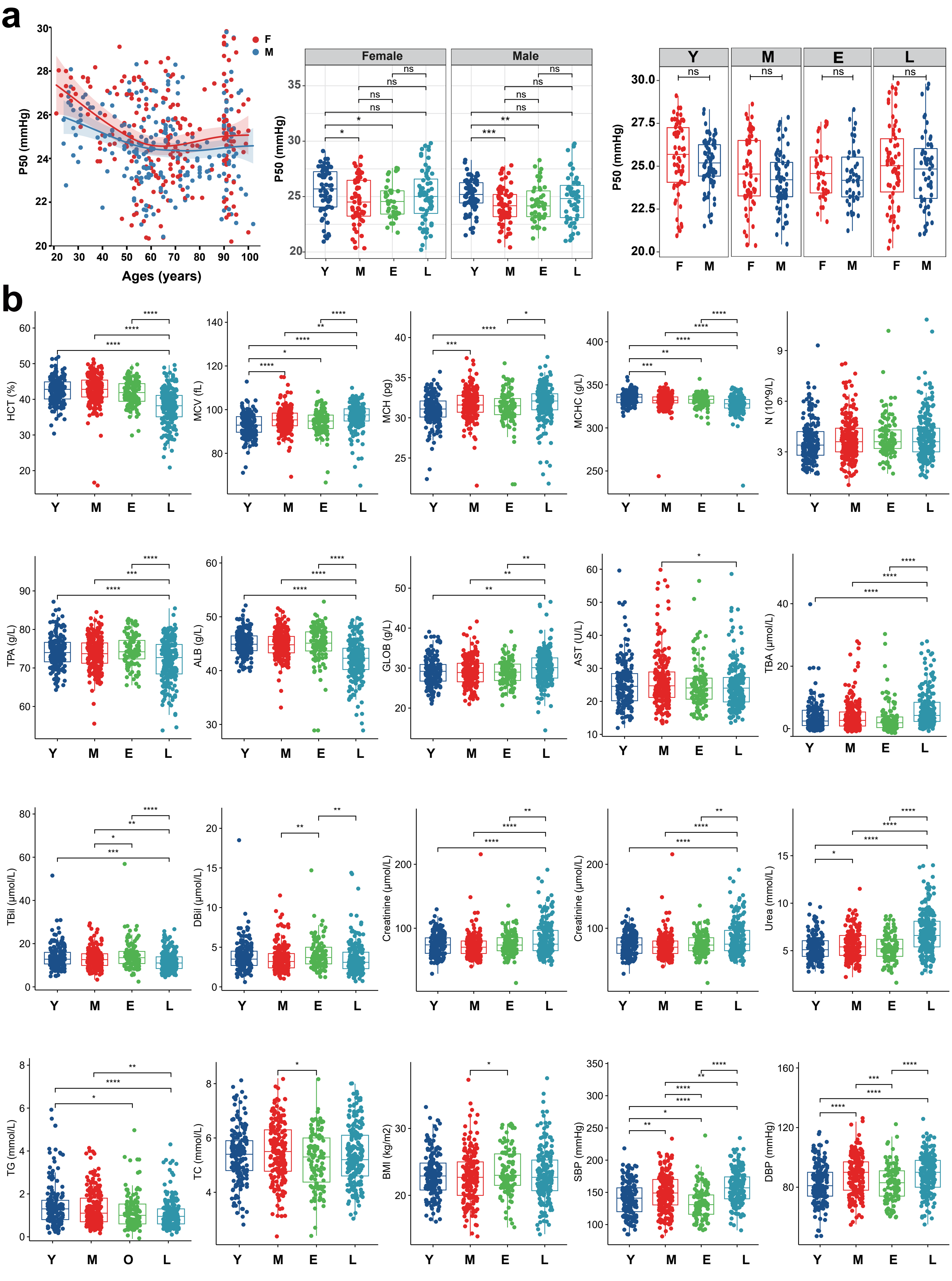
a
F
M
P50 (mmHg)
Ages (years)
Female
Male
Y
M
E
L
F
M
b
HCT (%)
MCV (fL)
MCH (pg)
MCHC (g/L)
N (10^9/L)
TPA (g/L)
ALB (g/L)
GLOB (g/L)
AST (U/L)
TBA (μmol/L)
TBil (μmol/L)
DBil (μmol/L)
Creatinine (μmol/L)
Creatinine (μmol/L)
Urea (mmol/L)
TG (mmol/L)
TC (mmol/L)
BMI (kg/m2)
SBP (mmHg)
DBP (mmHg)
Y
M
O
L

Fig.S1

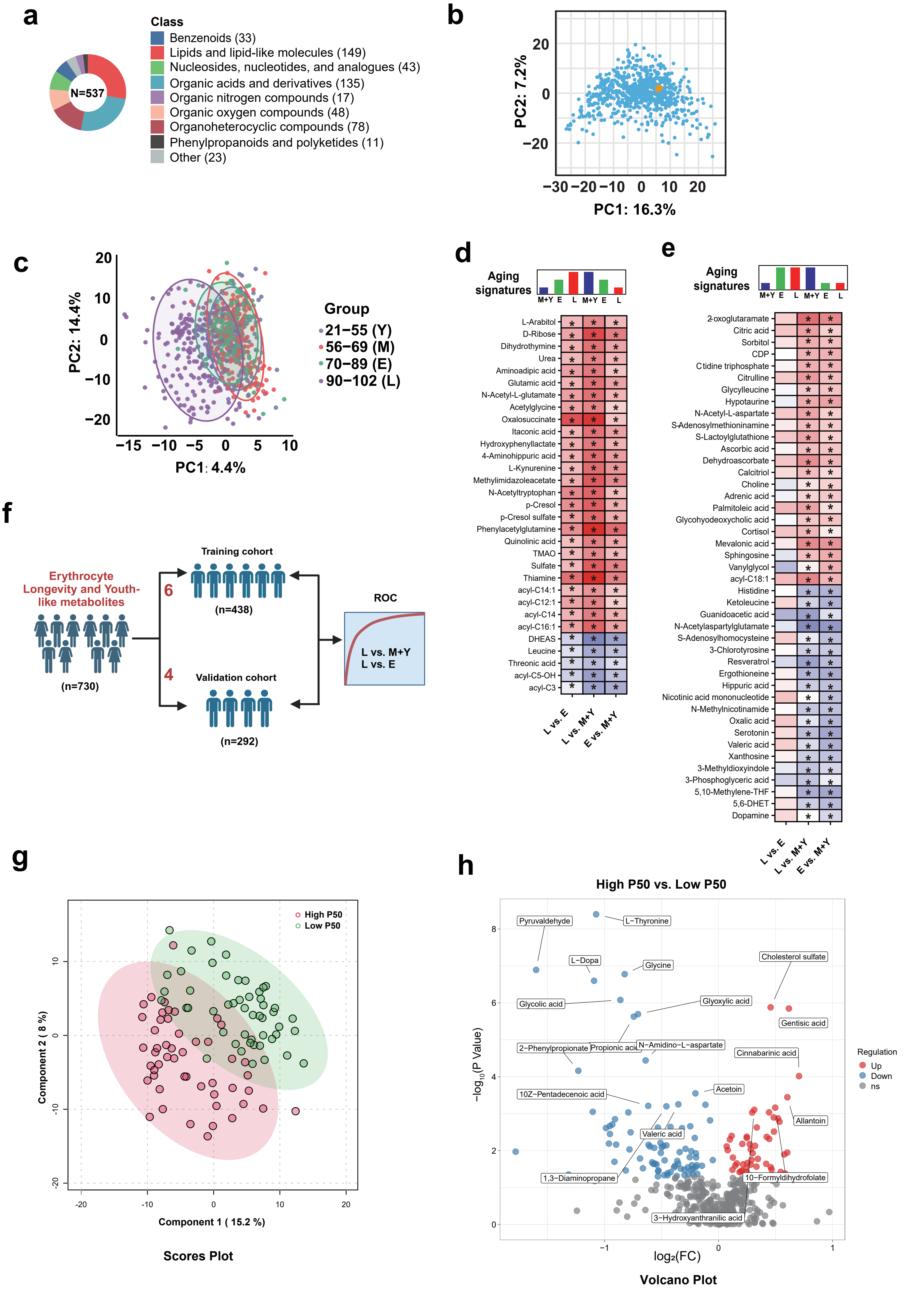
a
Class
Benzenoids (33)
Lipids and lipid-like molecules (149)
Nucleosides, nucleotides, and analogues (43)
Organic acids and derivatives (135)
Organic nitrogen compounds (17)
Organic oxygen compounds (48)
Organoheterocyclic compounds (78)
Phenylpropanoids and polyketides (11)
Other (23)
N=537
b
PC2: 7.2%
PC1: 16.3%
c
PC2: 14.4%
PC1: 4.4%
Group
21−55 (Y)
56−69 (M)
70−89 (E)
90−102 (L)
d
Aging signatures
M+Y E L M+Y E L
L-Arabitol
D-Ribose
Dihydrothymine
Urea
Aminoadipic acid
Glutamic acid
N-Acetyl-L-glutamate
Acetylglycine
Oxalosuccinate
Itaconic acid
Hydroxyphenyllactate
4-Aminohippuric acid
L-Kynurenine
Methylimidazoleacetate
N-Acetyltryptophan
p-Cresol
p-Cresol sulfate
Phenylacetylglutamine
Quinolinic acid
TMAO
Sulfate
Thiamine
acyl-C14:1
acyl-C12:1
acyl-C14
acyl-C16:1
DHEAS
Leucine
Threonic acid
acyl-C5-OH
acyl-C3
L vs. E
L vs. M+Y
E vs. M+Y
e
Aging signatures
M+Y E L M+Y E L
2-oxoglutaramate
Citric acid
Sorbitol
CDP
Ctidine triphosphate
Citrulline
Glycylleucine
Hypotaurine
N-Acetyl-L-aspartate
S-Adenosylmethioninamine
S-Lactoylglutathione
Ascorbic acid
Dehydroascorbate
Calcitriol
Choline
Adrenic acid
Palmitoleic acid
Glycohyodeoxycholic acid
Cortisol
Mevalonic acid
Sphingosine
Vanylglycol
acyl-C18:1
Histidine
Ketoleucine
Guanidoacetic acid
N-Acetylaspartylglutamate
S-Adenosylhomocysteine
3-Chlorotyrosine
Resveratrol
Ergothioneine
Hippuric acid
Nicotinic acid mononucleotide
N-Methylnicotinamide
Oxalic acid
Serotonin
Valeric acid
Xanthosine
3-Methyldioxyindole
3-Phosphoglyceric acid
5,10-Methylene-THF
5,6-DHET
Dopamine
L vs. E
L vs. M+Y
E vs. M+Y
f
Erythrocyte Longevity and Youth-like metabolites
(n=730)
Training cohort
6
(n=438)
4
Validation cohort
(n=292)
ROC
L vs. M+Y
L vs. E
g
High P50
Low P50
Component 2 (8 %)
Component 1 (15.2 %)
Scores Plot
h
High P50 vs. Low P50
Pyruvaldehyde
L-Thyronine
L-Dopa
Glycine
Cholesterol sulfate
Glycolic acid
Glyoxylic acid
Gentisic acid
2−Phenylpropionate
Propionic acid
N−Amidino−L−aspartate
Cinnabarinic acid
10Z−Pentadecenoic acid
Acetoin
Allantoin
Valeric acid
1,3−Diaminopropane
10−Formyldihydrofolate
3−Hydroxyanthranilic acid
−log10(P Value)
log2(FC)
Regulation
Up
Down
ns
Volcano Plot

Fig.S2

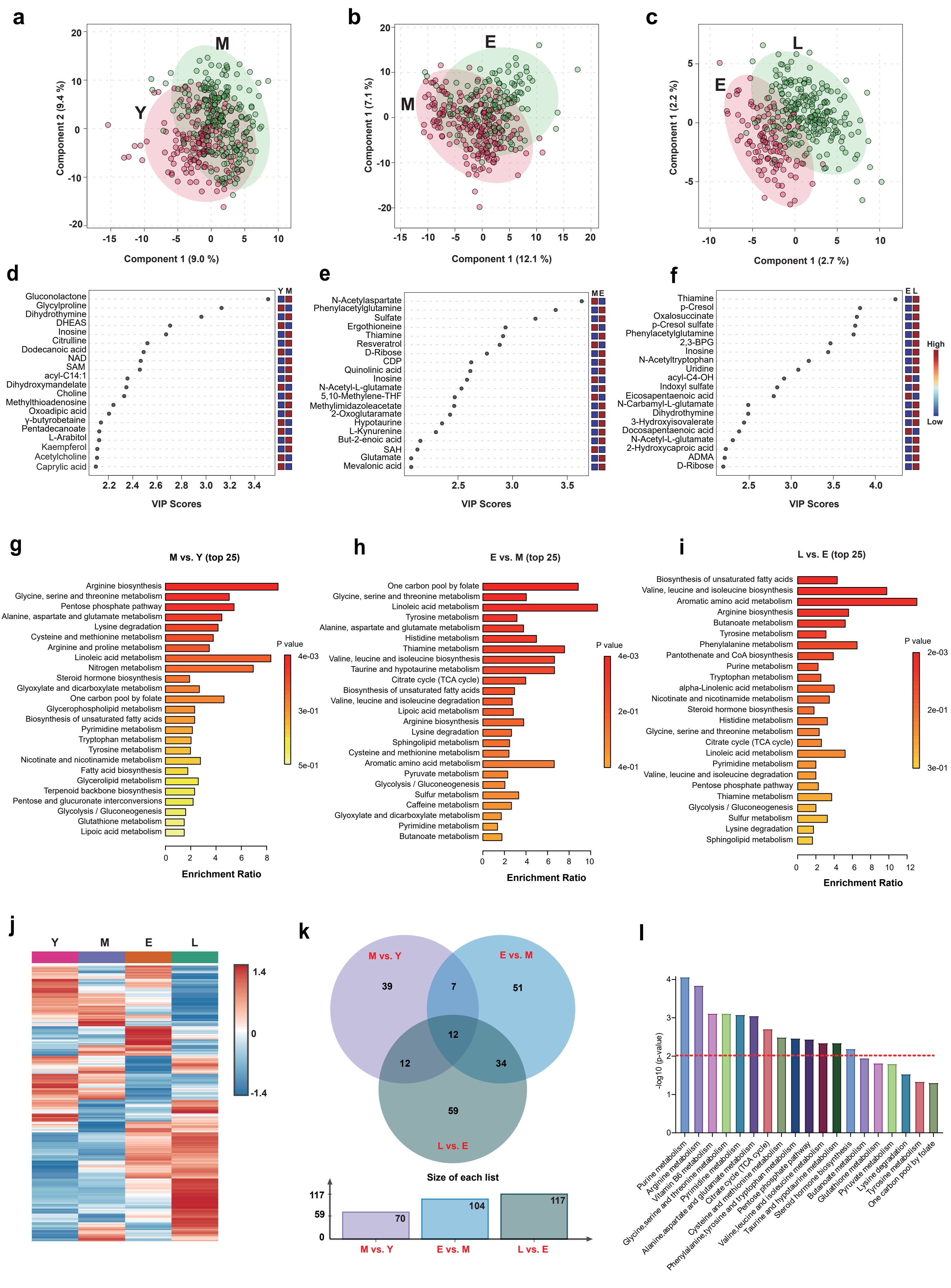
a
M
Y
Component 2 (9.4 %)
Component 1 (9.0 %)
b
E
M
Component 1 (7.1 %)
Component 1 (12.1 %)
c
L
E
Component 1 (2.2 %)
Component 1 (2.7 %)
d
Y M
Gluconolactone
Glycylproline
Dihydrothymine
DHEAS
Inosine
Citrulline
Dodecanoic acid
NAD
SAM
acyl-C14:1
Dihydroxymandelate
Choline
Methylthioadenosine
Oxoadipic acid
γ-butyrobetaine
Pentadecanoate
L-Arabitol
Kaempferol
Acetylcholine
Caprylic acid
VIP Scores
e
M E
N-Acetylaspartate
Phenylacetylglutamine
Sulfate
Ergothioneine
Thiamine
Resveratrol
D-Ribose
CDP
Quinolinic acid
Inosine
N-Acetyl-L-glutamate
5,10-Methylene-THF
Methylimidazoleacetate
2-Oxoglutaramate
Hypotaurine
L-Kynurenine
But-2-enoic acid
SAH
Glutamate
Mevalonic acid
VIP Scores
f
E L
Thiamine
p-Cresol
Oxalosuccinate
p-Cresol sulfate
Phenylacetylglutamine
2,3-BPG
Inosine
N-Acetyltryptophan
Uridine
acyl-C4-OH
Indoxyl sulfate
Eicosapentaenoic acid
N-Carbamyl-L-glutamate
Dihydrothymine
3-Hydroxyisovalerate
Docosapentaenoic acid
N-Acetyl-L-glutamate
2-Hydroxycaproic acid
ADMA
D-Ribose
VIP Scores
High
Low
g
M vs. Y (top 25)
Arginine biosynthesis
Glycine, serine and threonine metabolism
Pentose phosphate pathway
Alanine, aspartate and glutamate metabolism
Lysine degradation
Cysteine and methionine metabolism
Arginine and proline metabolism
Linoleic acid metabolism
Nitrogen metabolism
Steroid hormone biosynthesis
Glyoxylate and dicarboxylate metabolism
One carbon pool by folate
Glycerophospholipid metabolism
Biosynthesis of unsaturated fatty acids
Pyrimidine metabolism
Tryptophan metabolism
Tyrosine metabolism
Nicotinate and nicotinamide metabolism
Fatty acid biosynthesis
Glycerolipid metabolism
Terpenoid backbone biosynthesis
Pentose and glucuronate interconversions
Glycolysis / Gluconeogenesis
Glutathione metabolism
Lipoic acid metabolism
P value
4e-03
3e-01
5e-01
Enrichment Ratio
h
E vs. M (top 25)
One carbon pool by folate
Glycine, serine and threonine metabolism
Linoleic acid metabolism
Tyrosine metabolism
Alanine, aspartate and glutamate metabolism
Histidine metabolism
Thiamine metabolism
Valine, leucine and isoleucine biosynthesis
Taurine and hypotaurine metabolism
Citrate cycle (TCA cycle)
Biosynthesis of unsaturated fatty acids
Valine, leucine and isoleucine degradation
Lipoic acid metabolism
Arginine biosynthesis
Lysine degradation
Sphingolipid metabolism
Cysteine and methionine metabolism
Aromatic amino acid metabolism
Pyruvate metabolism
Glycolysis / Gluconeogenesis
Sulfur metabolism
Caffeine metabolism
Glyoxylate and dicarboxylate metabolism
Pyrimidine metabolism
Butanoate metabolism
P value
4e-03
2e-01
4e-01
Enrichment Ratio
i
L vs. E (top 25)
Biosynthesis of unsaturated fatty acids
Valine, leucine and isoleucine biosynthesis
Aromatic amino acid metabolism
Arginine biosynthesis
Butanoate metabolism
Tyrosine metabolism
Phenylalanine metabolism
Pantothenate and CoA biosynthesis
Purine metabolism
Tryptophan metabolism
alpha-Linolenic acid metabolism
Nicotinate and nicotinamide metabolism
Steroid hormone biosynthesis
Histidine metabolism
Glycine, serine and threonine metabolism
Citrate cycle (TCA cycle)
Linoleic acid metabolism
Pyrimidine metabolism
Valine, leucine and isoleucine degradation
Pentose phosphate pathway
Thiamine metabolism
Glycolysis / Gluconeogenesis
Sulfur metabolism
Lysine degradation
Sphingolipid metabolism
P value
2e-03
2e-01
3e-01
Enrichment Ratio
j
Y M E L
1.4
0
-1.4
k
M vs. Y
E vs. M
L vs. E
39
7
51
12
12
34
59
Size of each list
117
59
0
70
104
117
M vs. Y
E vs. M
L vs. E
l
-log10 (p-value)
Purine metabolism
Arginine metabolism
Vitamin B6 metabolism
Glycine,serine and threonine metabolism
Pyrimidine metabolism
Alanine,aspartate and glutamate metabolism
Citrate cycle (TCA cycle)
Cysteine and methionine metabolism
Phenylalanine,tyrosine and tryptophan metabolism
Pentose phosphate pathway
Valine,leucine and isoleucine metabolism
Taurine and hypotaurine metabolism
Steroid hormone biosynthesis
Butanoate metabolism
Glutathione metabolism
Pyruvate metabolism
Lysine degradation
Tyrosine metabolism
One carbon pool by folate

Fig.S3

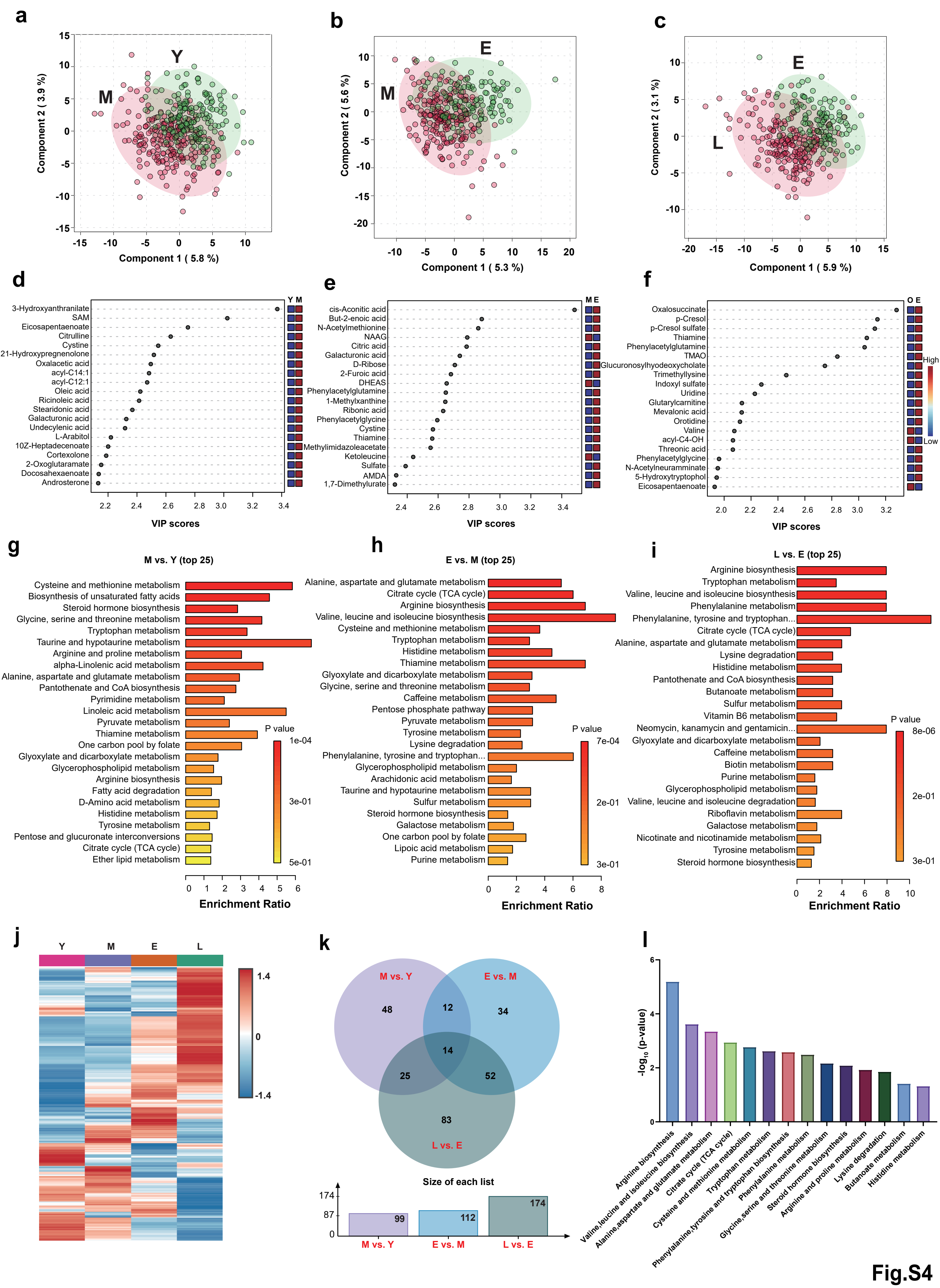

a
M
Y
Component 1 (5.8 %)
Component 2 (3.9 %)
b
M
E
Component 1 (5.3 %)
Component 2 (5.6 %)
c
L
E
Component 1 (5.9 %)
Component 2 (3.1 %)
d
Y M
3-Hydroxyanthranilate
SAM
Eicosapentaenoate
Citrulline
Cystine
21-Hydroxypregnenolone
Oxalacetic acid
acyl-C14:1
acyl-C12:1
Oleic acid
Ricinoleic acid
Stearidonic acid
Galacturonic acid
Undecylenic acid
L-Arabitol
10Z-Heptadecenoate
Cortexolone
2-Oxoglutaramate
Docosahexaenoate
Androsterone
VIP scores
e
M E
cis-Aconitic acid
But-2-enoic acid
N-Acetylmethionine
NAAG
Citric acid
Galacturonic acid
D-Ribose
2-Furoic acid
DHEAS
Phenylacetylglutamine
1-Methylxanthine
Ribonic acid
Phenylacetylglycine
Cystine
Thiamine
Methylimidazoleacetate
Ketoleucine
Sulfate
AMDA
1,7-Dimethylurate
VIP scores
f
O E
Oxalosuccinate
p-Cresol
p-Cresol sulfate
Thiamine
Phenylacetylglutamine
TMAO
Glucuronosylhyodeoxycholate
Trimethyllysine
Indoxyl sulfate
Uridine
Glutarylcarnitine
Mevalonic acid
Orotidine
Valine
acyl-C4-OH
Threonic acid
Phenylacetylglycine
N-Acetylneuramminate
5-Hydroxytryptophol
Eicosapentaenoate
High
Low
VIP scores
g
M vs. Y (top 25)
Cysteine and methionine metabolism
Biosynthesis of unsaturated fatty acids
Steroid hormone biosynthesis
Glycine, serine and threonine metabolism
Tryptophan metabolism
Taurine and hypotaurine metabolism
Arginine and proline metabolism
alpha-Linolenic acid metabolism
Alanine, aspartate and glutamate metabolism
Pantothenate and CoA biosynthesis
Pyrimidine metabolism
Linoleic acid metabolism
Pyruvate metabolism
Thiamine metabolism
One carbon pool by folate
Glyoxylate and dicarboxylate metabolism
Glycerophospholipid metabolism
Arginine biosynthesis
Fatty acid degradation
D-Amino acid metabolism
Histidine metabolism
Tyrosine metabolism
Pentose and glucuronate interconversions
Citrate cycle (TCA cycle)
Ether lipid metabolism
P value
1e-04
3e-01
5e-01
Enrichment Ratio
h
E vs. M (top 25)
Alanine, aspartate and glutamate metabolism
Citrate cycle (TCA cycle)
Arginine biosynthesis
Valine, leucine and isoleucine biosynthesis
Cysteine and methionine metabolism
Tryptophan metabolism
Histidine metabolism
Thiamine metabolism
Glyoxylate and dicarboxylate metabolism
Glycine, serine and threonine metabolism
Caffeine metabolism
Pentose phosphate pathway
Pyruvate metabolism
Tyrosine metabolism
Lysine degradation
Phenylalanine, tyrosine and tryptophan...
Glycerophospholipid metabolism
Arachidonic acid metabolism
Taurine and hypotaurine metabolism
Sulfur metabolism
Steroid hormone biosynthesis
Galactose metabolism
One carbon pool by folate
Lipoic acid metabolism
Purine metabolism
P value
7e-04
2e-01
3e-01
Enrichment Ratio
i
L vs. E (top 25)
Arginine biosynthesis
Tryptophan metabolism
Valine, leucine and isoleucine biosynthesis
Phenylalanine metabolism
Phenylalanine, tyrosine and tryptophan...
Citrate cycle (TCA cycle)
Alanine, aspartate and glutamate metabolism
Lysine degradation
Histidine metabolism
Pantothenate and CoA biosynthesis
Butanoate metabolism
Sulfur metabolism
Vitamin B6 metabolism
Neomycin, kanamycin and gentamicin...
Glyoxylate and dicarboxylate metabolism
Caffeine metabolism
Biotin metabolism
Purine metabolism
Glycerophospholipid metabolism
Valine, leucine and isoleucine degradation
Riboflavin metabolism
Galactose metabolism
Nicotinate and nicotinamide metabolism
Tyrosine metabolism
Steroid hormone biosynthesis
P value
8e-06
2e-01
3e-01
Enrichment Ratio
j
Y M E L
1.4
0
-1.4
k
M vs. Y
E vs. M
L vs. E
48 12 34 14 25 52 83
Size of each list
99 112 174
M vs. Y E vs. M L vs. E
l
-log10 (p-value)
Arginine biosynthesis
Valine,leucine and isoleucine biosynthesis
Alanine,aspartate and glutamate metabolism
Citrate cycle (TCA cycle)
Cysteine and methionine metabolism
Tryptophan metabolism
Phenylalanine,tyrosine and tryptophan biosynthesis
Phenylalanine metabolism
Glycine,serine and threonine metabolism
Steroid hormone biosynthesis
Arginine and proline metabolism
Lysine degradation
Butanoate metabolism
Histidine metabolism

Fig.S4

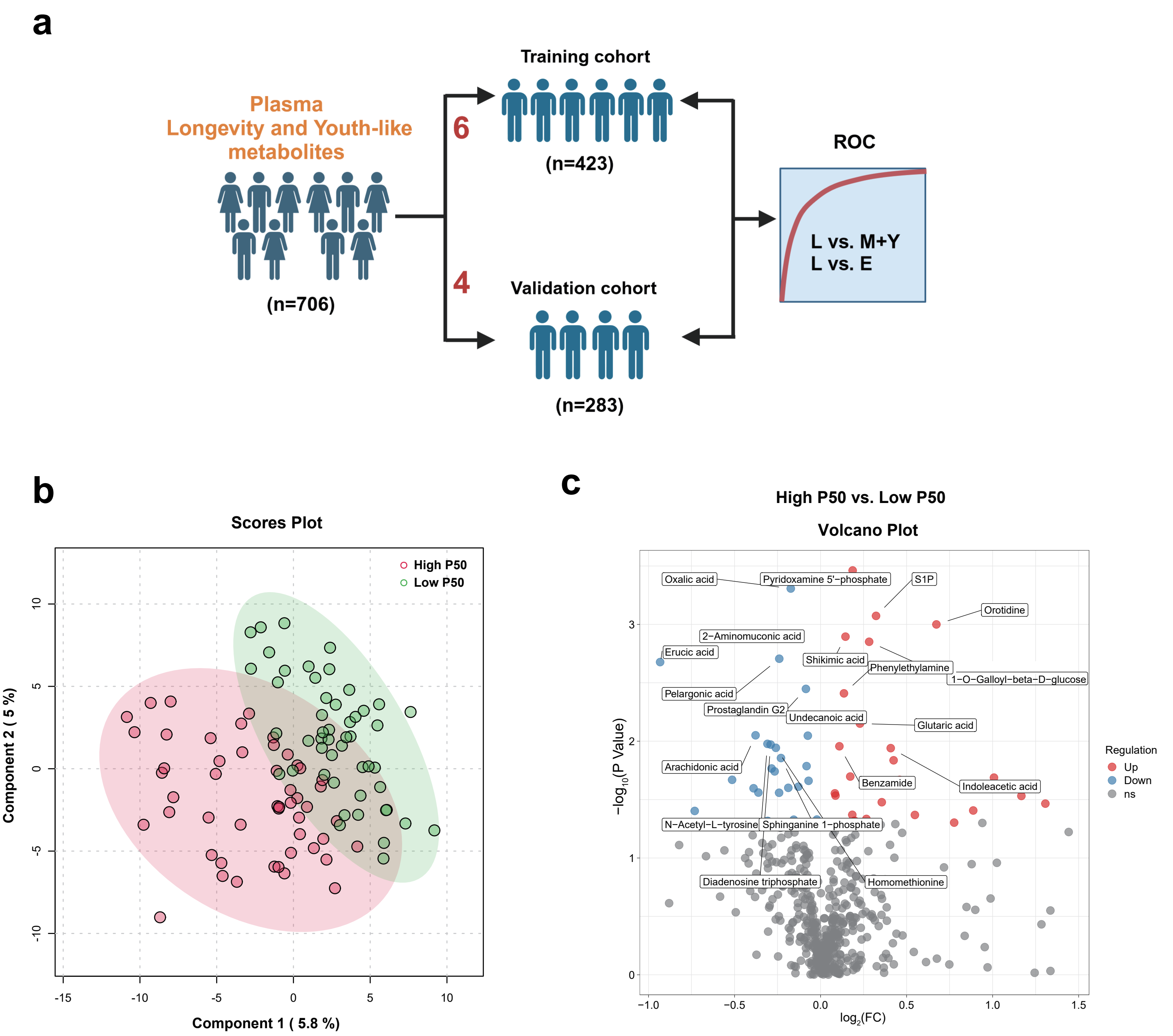
a
Training cohort
Plasma
Longevity and Youth-like
metabolites
6
(n=423)
ROC
L vs. M+Y
L vs. E
(n=706)
4
Validation cohort
(n=283)
b
Scores Plot
High P50
Low P50
Component 2 (5 %)
Component 1 (5.8 %)
c
High P50 vs. Low P50
Volcano Plot
Oxalic acid
Pyridoxamine 5'−phosphate
S1P
Orotidine
2−Aminomuconic acid
Erucic acid
Shikimic acid
Phenylethylamine
1−O−Galloyl−beta−D−glucose
Pelargonic acid
Prostaglandin G2
Undecanoic acid
Glutaric acid
Arachidonic acid
Benzamide
Indoleacetic acid
N−Acetyl−L−tyrosine
Sphinganine 1−phosphate
Diadenosine triphosphate
Homomethionine
−log10(P Value)
log2(FC)
Regulation
Up
Down
ns

Fig. S5

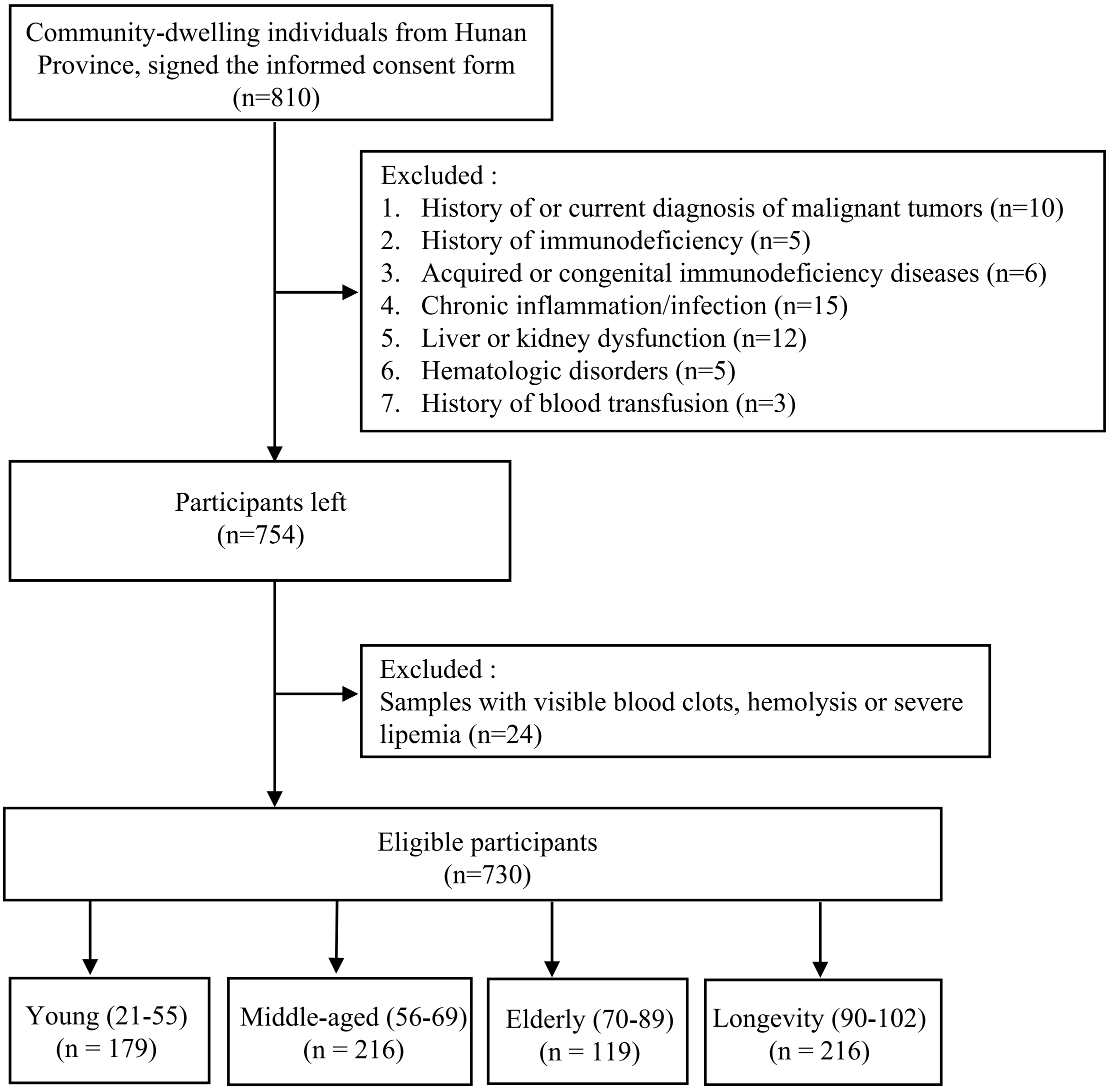
Community-dwelling individuals from Hunan Province, signed the informed consent form (n=810)
Excluded :
1. History of or current diagnosis of malignant tumors (n=10)
2. History of immunodeficiency (n=5)
3. Acquired or congenital immunodeficiency diseases (n=6)
4. Chronic inflammation/infection (n=15)
5. Liver or kidney dysfunction (n=12)
6. Hematologic disorders (n=5)
7. History of blood transfusion (n=3)
Participants left (n=754)
Excluded : Samples with visible blood clots, hemolysis or severe lipemia (n=24)
Eligible participants (n=730)
Young (21-55) (n = 179)
Middle-aged (56-69) (n = 216)
Elderly (70-89) (n = 119)
Longevity (90-102) (n = 216)

Fig.S6

Table S1

Aging and Longevity Population Information Collection Form

- **Participant Code (Num): ______**
- **Date (Date):**<yyyy/mm/dd>
- **Residence (Residence): ______** Town ______ Village

A. General Information

- **Name: ____________**
- **Gender:** 1 Male 2 Female
- **Date of Birth:** <yyyy/mm/dd>
- **Ethnicity (Nation): __________**
- **Occupation (Work): __________**
- **Marital Status: __________**
- **Educational Level (Degree):** 1 Illiterate 2 Primary School 3 Middle School 4 High School 5 Associate Degree 6 Bachelor's Degree
- **ID Number (ID):** ######-########-###___
- **Contact Phone (Phone):** ① ### - #### - ####

B. Medical History

1. Cardiovascular Diseases: ① No, ② Yes: __________________
2. Digestive, Respiratory, Urinary Diseases: ① No, ② Yes: __________________
3. History of Brain Injury, Coma, Seizures, Intracranial Infection: ① No, ② Yes: __________________
4. Infectious Diseases: ① No, ② Yes: __________________
5. Psychiatric Disorders or Others:① No, ② Yes: __________________
6. History of Trauma or Surgery: ① No, ② Yes: __________________
7. Drug Allergies: ① No, ② Yes: __________________
8. Family History of Hereditary Diseases: ① No, ② Yes: __________________
9. Long-term Medication History: ① No, ② Yes: __________________
10. Medication Taken for Specific Diseases (multiple selections possible): ######### ① Cardiovascular Disease ② Neurological Disorders ③ Digestive and Respiratory ④ Infectious Diseases ⑤ Others: __________________

C. Physical Examination

- **Height (H):** ### (cm)
- **Weight (W):** ### (kg)
- **Waist Circumference (Waist):** ### cm
- **Blood Pressure (Bp):** ###/### mmHg
- **Body Temperature (Tem):** ### (°C)
- **Pulse (Pulse):** ### beats/min

- **Heart Function Normal (Heart):** ① Yes, ② No: __________________
- **Lung Function Normal (Lung):** ① Yes, ② No: __________________

D. Gynecological Indicators

1. Age at Menarche: ### years;
2. Regular Menstrual Cycle: ① Yes, ② No;
3. Menopause Status: ① Yes, ② No;
4. Age at Menopause: ### years;

E. Hospitalization Records

1. Reason for Hospitalization: ___________________________;
2. Admission Date: @<yyyy/mm/dd>;
3. Family History of This Disease: ① Yes, ② No;
   If yes, Relationship to You: _________, Current Age: ### years, Age at Onset: ### years;

F. Lifestyle

1. Weekly Alcohol Consumption: ① No, ② Yes;
   1.1 Drinking Liquor: For ### years, ### ounces per week, Abstinent for ### years;
   1.2 Drinking Beer: For ### years, ### bottles per week, Abstinent for ### years;
2. Smoking Status: ① Yes, Smoking ### cigarettes per day, Smoking for ### years, ② No, Never Smoked or Quit Smoking ### years ago;
3. Daily Sedentary Time: ① 1.5 hours, ② 1.5 - 2.5 hours, ③ 2.5 - 3.5 hours, ④ 3.5 - 4.5 hours, ⑤ ≥4.5 hours;
4. Sleep Duration: ① Less than 6 hours/day, ② 6-9 hours/day, ③ More than 9 hours/day;
5. Lifestyle Habits: ① Mostly Indoors, ② Daily Outdoor Walks;

G. Dietary Habits

1. Your Dietary Preference: ① Sweet, ② Salty, ③ Spicy, ④ Mildly Spicy, ⑤ Light.
2. Frequency of consuming dairy products : ① Never, ② Once a month, ③ 1-3 days/week, ④ 4-6 days/week, ⑤ Every day.
3. Frequency of consuming desserts: ① Never, ② Once a month, ③ 1-3 days/week, ④ 4-6 days/week, ⑤ Every day.
4. Frequency of consuming pickled vegetables: ① Never, ② Once a month, ③ 1-3 days/week, ④ 4-6 days/week, ⑤ Every day.
5. Frequency of consuming eggs: ① Never, ② Once a month, ③ 1-3 days/week, ④ 4-6 days/week, ⑤ Every day.
6. Frequency of consuming fish and seafood: ① Never, ② Once a month, ③ 1-3 days/week, ④ 4-6 days/week, ⑤ Every day.
7. Frequency of consuming fresh vegetables: ① Never, ② Once a month, ③ 1-3 days/week, ④ 4-6 days/week, ⑤ Every day.

8. Frequency of consuming fried foods: ① Never, ② Once a month, ③ 1-3 days/week, ④ 4-6 days/week, ⑤ Every day.
9. Frequency of consuming fruits: ① Never, ② Once a month, ③ 1-3 days/week, ④ 4-6 days/week, ⑤ Every day.
10. Frequency of consuming beans and bean products: ① Never, ② Once a month, ③ 1-3 days/week, ④ 4-6 days/week, ⑤ Every day.
11. Frequency of consuming nuts: ① Never, ② Once a month, ③ 1-3 days/week, ④ 4-6 days/week, ⑤ Every day.
12. Frequency of consuming poultry: ① Never, ② Once a month, ③ 1-3 days/week, ④ 4-6 days/week, ⑤ Every day.
13. Frequency of consuming red meat (pork, beef, lamb, rabbit, etc.): ① Never, ② Once a month, ③ 1-3 days/week, ④ 4-6 days/week, ⑤ Every day.
14. Frequency of drinking tea: ① Never, ② Once a month, ③ 1-3 days/week, ④ 4-6 days/week, ⑤ Every day.
15. Predominantly used fats or oils: ① Animal oil, ② Vegetable oil, ③ Both.
16. Frequency of consuming rice: ① Never, ② Once a month, ③ 1-3 days/week, ④ 4-6 days/week, ⑤ Every day.
17. Frequency of consuming flour-based foods: ① Never, ② Once a month, ③ 1-3 days/week, ④ 4-6 days/week, ⑤ Every day.
