# Supplementary material for: Longevity Humans Have Youthful Erythrocyte Function and Metabolic Signatures
Source: Aging Cell. 2025 Feb 9;24(5):e14482. doi: 10.1111/acel.14482 (PMC12074018; doi:10.1111/acel.14482)
Supplement: Supplementary file 1 — Figure S1. Sexual difference of erythrocyte function in age groups and other clinical variables comparisons between groups. a, Comparison of P50 between females and males in different age groups. b, Boxplots for comparing clinical variables between groups. HCT, hematocrit; MCV, mean corpuscular volume; MCH, mean corpuscular hemoglobin; MCHC, mean corpuscular hemoglobin concentration; N, neutrophil; AST, aspartate aminotransferase; TPA, total serum protein; ALB, albumin; GLOB, globulin; AGR, albumin–globulin ratio; TBil, total bilirubin; DBil, direct bilirubin; TBA, total bile acid; eGFR, estimated glomerular filtration rate; TG, triglyceride; TC, total cholesterol; BMI, body mass index; SBP, systolic blood pressure; DBP, diastolic blood pressure. Data are mean ± s.d. ****p < 0.0001, ***p < 0.001, **p < 0.01, *p < 0.05; Kruskal–Wallis with Dunn’s test; ns, not significant. Figure S2. Erythrocyte metabolomics analysis and the aging signatures. a, Chemical composition of erythrocyte metabolites using the super class from HMDB database. b, Principal component analysis (PCA) of the erythrocyte metabolome of 730 participants and corresponding Quality Control samples (QC, labeled orange). QC samples are highly clustered, demonstrating low technical variance and high metabolomic reliability, SERRF correction method for batch correction. c, The partial least squares discriminant analysis (PLSDA) plot between different age groups. d‐e, Changes in the relative abundance of erythrocyte metabolites between the longevity, elderly, middle‐aged, and young groups according to aging signatures of differential abundance. The aging signatures included taxa whose abundance was increased or decreased with age. f, Flowchart illustrating the construction of a machine learning model based on erythrocyte longevity and youth‐like metabolites. g, PLSDA plot showing the metabolic differences in erythrocytes between the high P50 and low P50 subgroups within the longevity group. h, Volcano plot d [file ACEL-24-e14482-s001.zip › Legends.docx]

**Extended Data Fig. S1 Sexual difference of erythrocyte function in age groups and other clinical variables comparisons between groups**

**a**, Comparison of P50 between females and males in different age groups.

**b**, Boxplots for comparing clinical variables between groups. HCT, hematocrit; MCV, mean corpuscular volume; MCH, mean corpuscular hemoglobin; MCHC, mean corpuscular hemoglobin concentration; N, neutrophil; AST, aspartate aminotransferase; TPA, total serum protein; ALB, albumin; GLOB, globulin; AGR, albumin-globulin ratio; TBil, total bilirubin; DBil, direct bilirubin; TBA, total bile acid; eGFR, estimated glomerular filtration rate; TG, triglyceride; TC, total cholesterol; BMI, body mass index; SBP, systolic blood pressure; DBP, diastolic blood pressure. Data are mean ± s.d. *****P*<0.0001, ****P*<0.001, ***P*<0.01, **P*<0.05; Kruskal-Wallis with Dunn’s test; ns, not significant.

**Extended Data Fig. S2 Erythrocyte metabolomics analysis and the aging signatures**

**a**, Chemical composition of erythrocyte metabolites using the super class from HMDB database.

**b**, Principal component analysis (PCA) of the erythrocyte metabolome of 730 participants and corresponding Quality Control samples (QC, labeled orange). QC samples are highly clustered, demonstrating low technical variance and high metabolomic reliability, SERRF correction method for batch correction.

**c**, The partial least squares discriminant analysis (PLSDA) plot between different age groups.

**d-e**, Changes in the relative abundance of erythrocyte metabolites between the longevity, elderly, middle-aged, and the young group according to aging signatures of differential abundance. The aging signatures included taxa whose abundance was increased or decreased with age.

**f,** Flowchart illustrating the construction of a machine learning model based on erythrocyte longevity and youth-like metabolites.

**g,** PLS-DA plot showing the metabolic differences in erythrocytes between the high P50 and low P50 subgroups within the longevity group.

**h,** Volcano plot depicting the metabolic differences in erythrocytes between the high P50 and low P50 subgroups within the longevity group.

**Extended Data Fig. S3 Erythrocyte metabolomics analysis for all the samples**

**a**, PLSDA plot showing metabolic features of Y (red) and M (green) group.

**b**, PLSDA plot showing metabolic features of M (red) and E (green) group.

**c**, PLSDA plot showing metabolic features of E (red) and L (green) group.

**d-f**, VIP values and ranking of top 20 molecules according to PLSDA analysis of different age group comparisons.

**g-i**, KEGG pathway enrichment analysis based on differentially abundant metabolites between groups.

**j**, Overall heatmap of all metabolites.

**k**, Venn diagram showing the overlap of differentially expressed erythrocyte metabolites between different groups.

**l**, The KEGG pathway analysis of all differential metabolites between groups.

**Extended Data Fig. S4 Plasma metabolomics analysis for all the samples**

**a**, PLSDA plot showing metabolic features of Y (red) and M (green) group.

**b**, PLSDA plot showing metabolic features of M (red) and E (green) group.

**c**, PLSDA plot showing metabolic features of E (red) and L (green) group.

**d-f**, VIP values and ranking of top 20 molecules according to PLSDA analysis of different age group comparisons.

**g-i**, KEGG pathway enrichment analysis based on differentially abundant metabolites between groups.

**j**, Overall heatmap of all metabolites.

**k**, Venn diagram showing the overlap of differentially expressed plasma metabolites between different groups.

**l**, The KEGG pathway analysis of all differential metabolites between groups.

**Extended Data Fig. S5 Plasma metabolomics analysis for High P50 versus Low P50 group within longevity individuals**

**a**, Flowchart illustrating the construction of a machine learning model based on plasma longevity and youth-like metabolites.

**b**, PLS-DA plot showing the metabolic differences in plasma between the high P50 and low P50 subgroups within the longevity group.

**c**, Volcano plot depicting the metabolic differences in plasma between the high P50 and low P50 subgroups within the longevity group.

**Extended Data Fig. S6 Flowchart of participant selection**

**Extended Data Table S1 Detailed questionnaire for assessing health conditions**
